# A Perioperative Paradigm of Cranioplasty With Polyetheretherketone: Comprehensive Management for Preventing Postoperative Complications

**DOI:** 10.3389/fsurg.2022.856743

**Published:** 2022-03-21

**Authors:** Zhenghui He, Yuxiao Ma, Chun Yang, Jiyuan Hui, Qing Mao, Guoyi Gao, Jiyao Jiang, Junfeng Feng

**Affiliations:** ^1^Brain Injury Center, Renji Hospital, School of Medicine, Shanghai Jiao Tong University, Shanghai, China; ^2^Shanghai Institute of Head Trauma, Shanghai, China; ^3^Department of Neurosurgery, Shanghai General Hospital, School of Medicine, Shanghai Jiao Tong University, Shanghai, China

**Keywords:** cranioplasty, polyetheretherketone, perioperative management, complication, subcutaneous effusion, infection

## Abstract

**Introduction:**

At present, lots of studies have discussed the effects and outcomes of cranioplasty using polyetheretherketone (PEEK). However, interventions or management for PEEK cranioplasty got less attention. This article presented a perioperative paradigm for preventing postoperative complications.

**Materials and Methods:**

Modified PEEK plates with certified safety were implanted in patients who received evolving perioperative paradigm. Serial perioperative managements were developed as a comprehensive paradigm to prevent correlated risk factors of postoperative complications, which mainly included managements of epidural collections and wound healing. The preparation of the surgical area and systemic state were essential before surgery. During the operation, the blood supply of the incision and the handling of dura and temporalis were highlighted in our paradigm. After cranioplasty, management of subcutaneous drainage and wound healing were stressed. Patients received conventional management from February 2017 to August 2018 in our center. After the evolving paradigm developed, patients received comprehensive perioperative management from September 2018 to August 2020.

**Results:**

A total of 104 patients who underwent PEEK cranioplasty were consecutively enrolled; 38 (36.5%) received conventional perioperative management, and 66 (63.5%) received evolving perioperative paradigm. The general information of the two groups was comparable. Notably, patients who received the evolving paradigm presented a significantly decreased incidence of postoperative complications from 47.4 to 18.2% (*P* < 0.01), among which the incidences of subcutaneous effusion, epidural hematoma, and subcutaneous infection decreased significantly.

**Conclusion:**

The evolving perioperative paradigm could effectively prevent risk factors and reduce related complications. It was valuable to promote these comprehensive managements and inspire more clinical practice on improving patients' outcomes after PEEK cranioplasty.

## Introduction

Cranioplasty is often desirable when patients recover after craniectomy to achieve better outcomes (1–5). Polyetheretherketone (PEEK) is a relatively fashion artificial material employed for cranioplasty (6). Compared with other materials, PEEK is considered the best nonmetal polymer and thus has been increasingly applied in clinical practice (2, 7, 8). Its firmness, lightness, and flexibility allow it to be three-dimensionally printed and implanted to repair complex cranial structures, contributing to its preferential use in cranioplasty (7–10).

However, PEEK implants are also associated with a variety of postoperative complications, including epidural collections (e.g., hemorrhage or effusion) and poor healing (e.g., wound infection or implant exposure) (11, 12), all of which are undesirable and can impair the surgical outcome. Postoperative hemorrhage is considered a serious complication, with an incidence of 5% (11–13), and can lead to a second operation or even death. Subcutaneous effusion, with an incidence of 8.1–22.6% (13–16), seems negligible, but can bring constant discomfort to patients and may even result in implant removal. Infection in the operative region is another common complication, closely related to implant failure (12, 15, 17, 18).

At present, lots of studies have reviewed the effects and outcomes of different materials for cranioplasty (19), including PEEK, titanium, and other artificial polymers (12, 13, 15). However, no study has developed the interventions or management for complications after PEEK cranioplasty. Therefore, in this study, we discussed a perioperative paradigm of PEEK cranioplasty. We compared the incidence of complications in patients receiving this paradigm and in those who received conventional management. Taken together, we presented comprehensive management as a novel perspective for preventing postoperative complications in PEEK cranioplasty.

## Materials and Methods

### Clinical Cases

**Figure 1:**
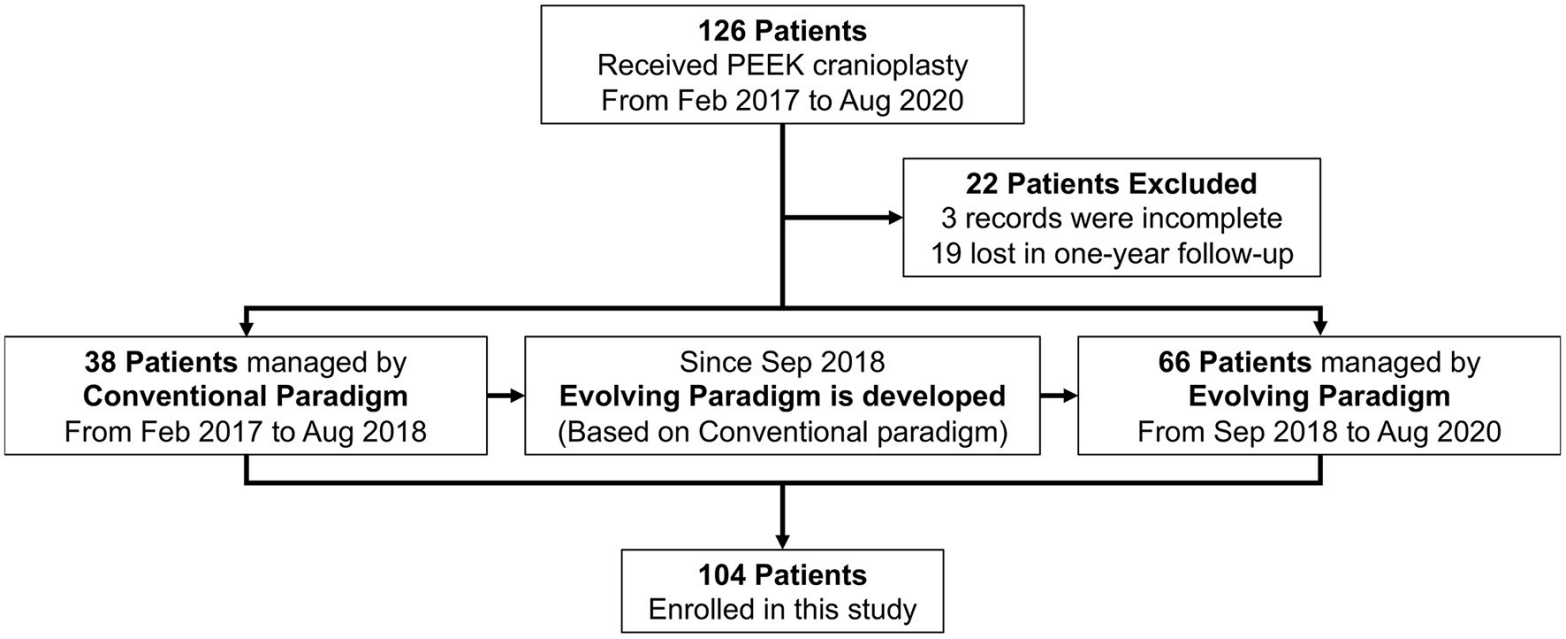
Patient flow chart. PEEK, Polyetheretherketone.

We consecutively collected patients who underwent PEEK cranioplasty in our department from February 2017 to August 2020 (Figure 1). Patients were followed up after 1 year postoperatively. The informed consent about collecting personal data was taken during follow-up. Conventional management was implemented for patients undergoing PEEK cranioplasty between February 2017 and August 2018. Based on the conventional perioperative procedure, modifications were introduced to form the evolving perioperative paradigm since September 2018. And patients who received the evolving paradigm between September 2018 and August 2020 were collected as the evolving group. This study was registered on ClinicalTrials.gov (NCT04707404). All modifications in our perioperative management were fully acknowledged and consented to by patients.

**Figure 2:**
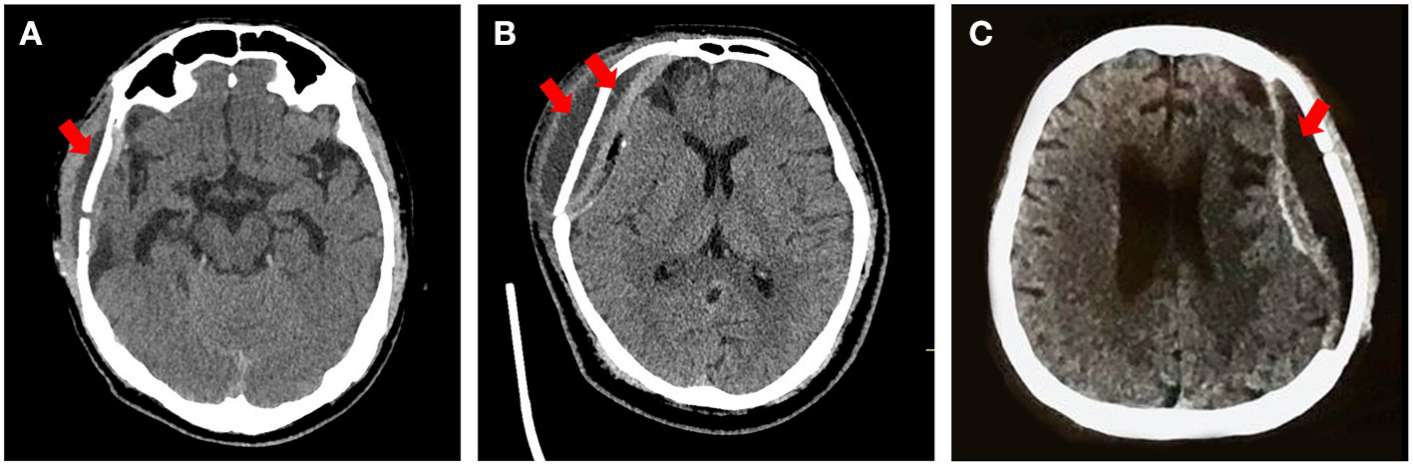
Typical types of effusion after PEEK cranioplasty. Red arrow: the effusion presents as a low-density area under the scalp on CT. **(A)** Subcutaneous effusion mainly outside the plate. **(B)** Subcutaneous effusion complicated with epidural effusion. **(C)** Effusion only at the epidural space.

**Figure 3:**
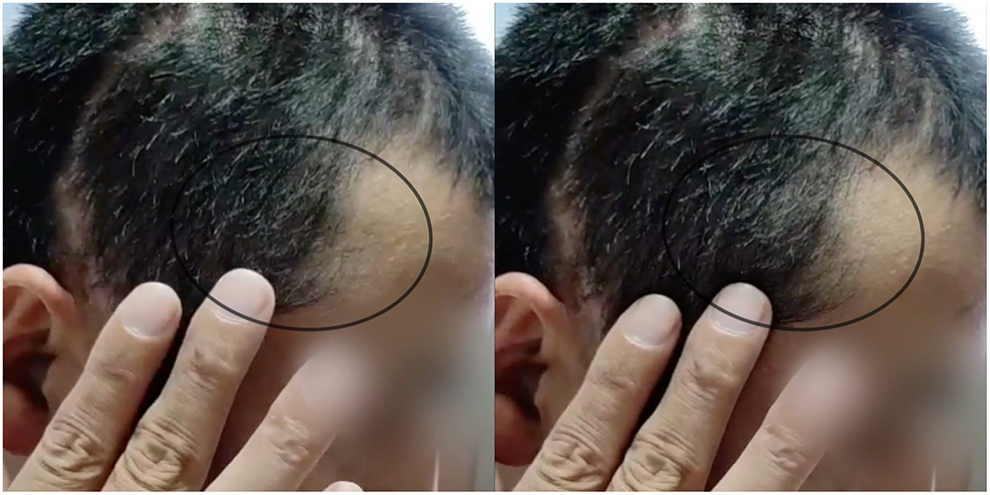
The appearance of subcutaneous effusion in physical examination. Black circle: the increasingly bulging scalp under pressing. (Consent for publication has taken from this patient).

The general information of enrolled patients was directly exported from the electronic medical system. The preoperative CT images were reconstructed to define the size and site of the skull defects. Complications that arose within 1 year postoperatively were retrieved from the patients' history records, follow-up survey, and postoperative CT images. Subcutaneous effusion was defined as fluid (low-density area) in the space outside and/or under the plate, diagnosed according to CT images (Figure 2) and physical examination (Figure 3, Supplementary Video 1).

### Modifications on the PEEK Plate

**Figure 4:**
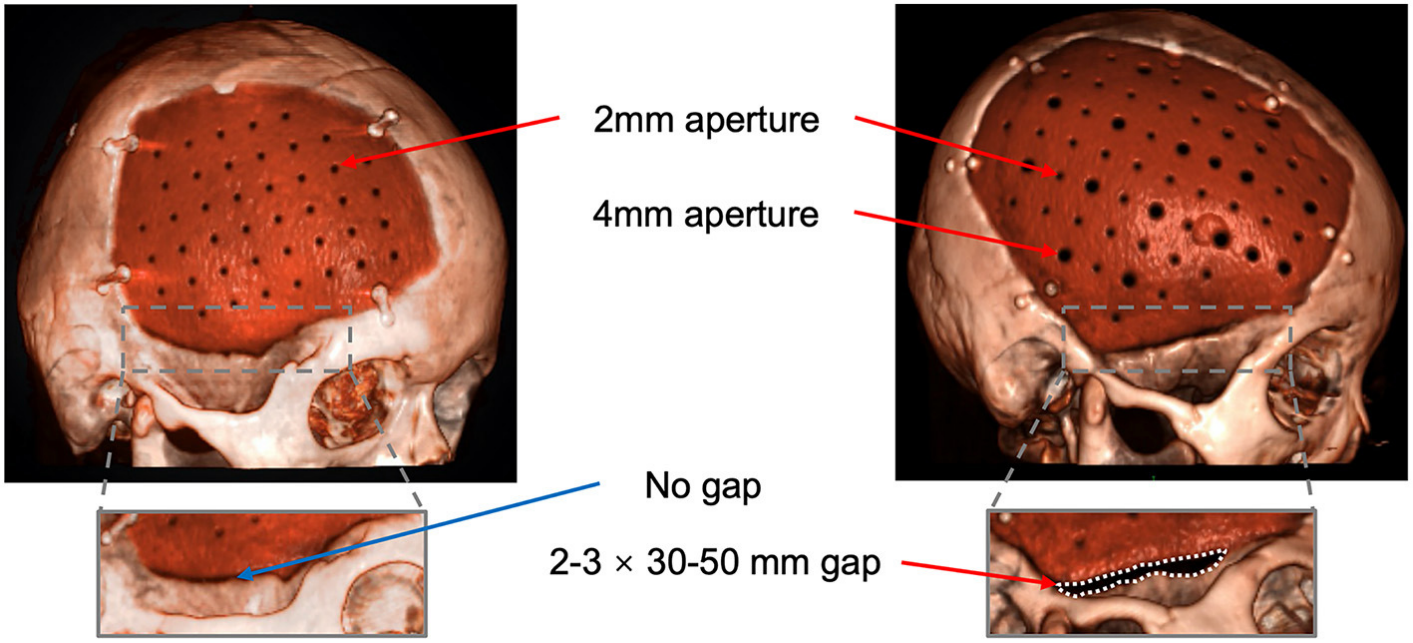
Reconstruction with the improved design of the PEEK plate. Arrows: the 2-mm and 4-mm apertures; Dashed line area: the gap between the plate and the temporal bone.

Before August 2018, patients implanted conventional PEEK plate, with 2 mm apertures and no temporal gap. After that, the PEEK plate was modified to improve the conduction between subcutaneous and epidural. As shown in Figure 4, the modified PEEK plate leaves a gap at the site of the base of the temporalis muscle, which is normally 2–3 mm in width and 3–5 cm in length. The gap on our modified plate was added during manufacturing. The apertures on the PEEK plate were modified to diameters of 2 and 4 mm and separated by a distance of 12 mm. As larger apertures may influence the strength of the PEEK plate, finite element mechanical analysis was then performed by a third-party laboratory to evaluate the strength of the modified plates, including maximum displacement, stress, and strain. The elasticity coefficient was calculated from stress and strain (see Supplementary Material).

### Comprehensive Perioperative Managements for PEEK Cranioplasty

**Figure 5:**
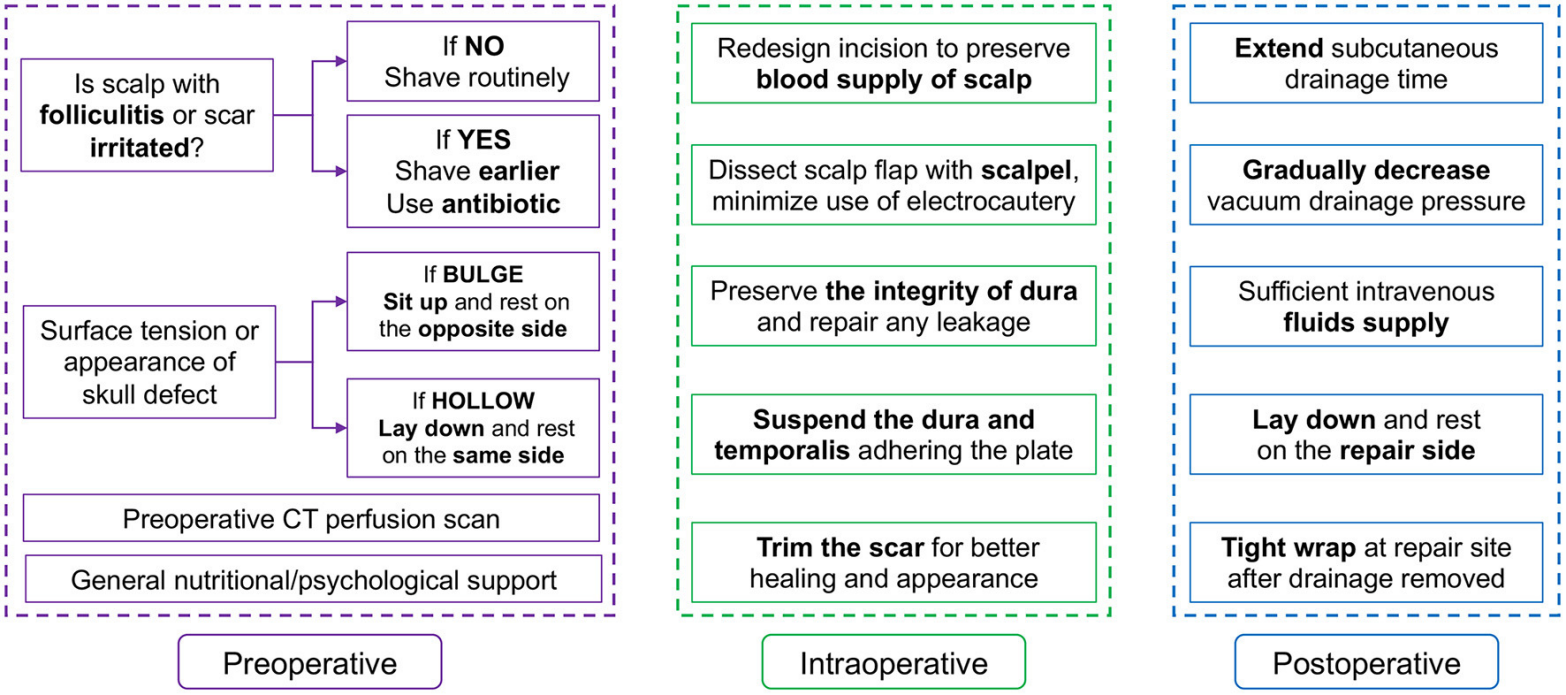
Summary of comprehensive perioperative managements.

PREOPERATIVE (Purple part in Figure 5):

The preoperative part emphasized the preparation of the surgical area and systemic state.

**Figure 6:**
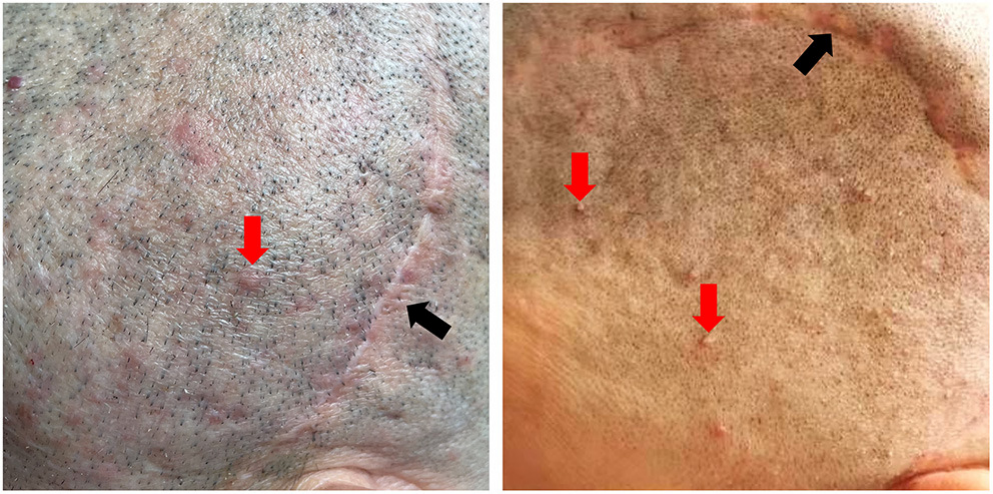
Patient's appearance of unfavorable preoperative scar and scalp. Red arrow: folliculitis around the surgical area; black arrow: irritated scar. (Consent for publication has taken from patients).

(1) Patients were shaved to better expose the possible folliculitis or irritated scars 4–7 days before surgery. For patients with folliculitis or irritated scars (Figure 6), antibiotics were also used topically in the surgical area or even systematically for 1 week, depending on the condition of the scalp. On the day before surgery, the scalp was shaved again.

(2) One week before surgery, some patients were asked to lay down on the repair side or were transfused additionally for rebounding. Some patients needed dehydrant or to sit up to relieve skin tension. For patients with ventricular shunt, the pressure threshold of the shunt valve could be switched down.

(3) If local health/insurance policy allowed, preoperative CT perfusion or CT angiography was recommended to precisely reveal the changes in the cerebrovasculature after recovery from disease.

(4) Nutrition and psychological supports were introduced for better preoperative state and postoperative recovery.

INTRAOPERATIVE (Green part in Figure 5):

The intraoperative part highlighted the blood supply of incision and handling of dura and temporalis.

**Figure 7:**
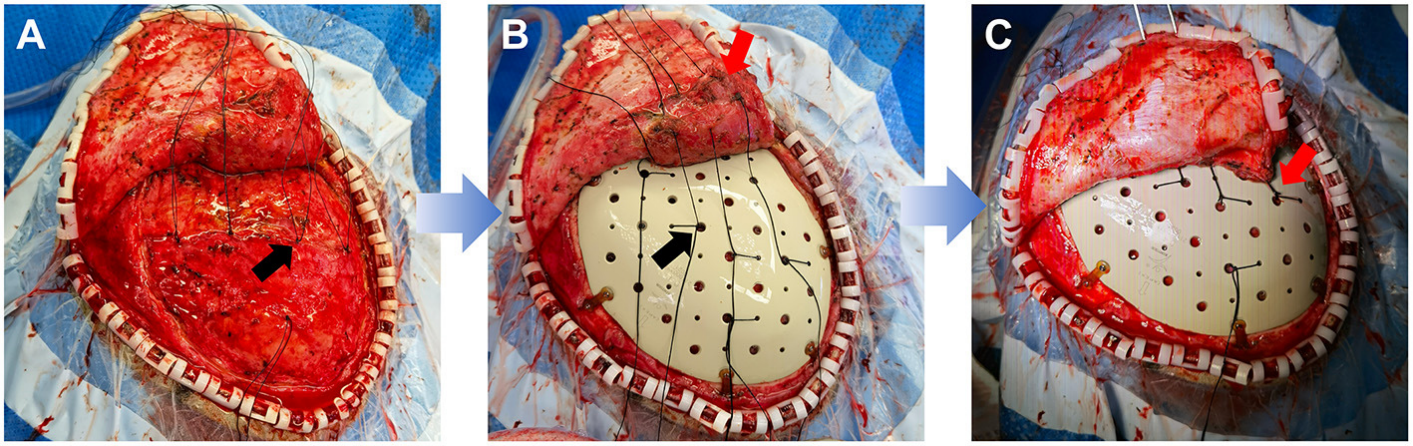
The procedure of suspending dura and temporalis. **(A)** Leaving the sutures on residues of temporalis fascia on dura. Black arrow: the tie of sutures on residues of temporalis fascia. **(B)** Suspending the dura on the plate and suturing the rim of the temporalis. Black arrow: the tie of suspending sutures on the plate; Red arrow: the sutures on the rim of temporalis. **(C)** Suspending the temporalis on the plate and tightly adhering the plate together with dura. Red arrow: the tie of suspending suture of both dura and temporalis. (Consent for publication has taken from this patient).

(1) The skin incision should be designed to protect the scalp blood supply as much as possible.

(2) The traditional scalpel was recommended over electrocautery to dissect the skin flap during the operation. Even if the electrocautery was necessary, the power was limited under 10–15 Watt to minimize the heat and electric stimulation.

(3) The integrity of the dura was preserved to the greatest extent possible while dissecting. Every leakage of dura should be tightly repaired. For large defect difficult to suture, covering temporalis fascia or artificial dura to repair was recommended.

(4) Before the plate was fixed, sutures were left on residues of temporalis fascia on dura for suspending (Figure 7A). After the plate was implanted, the dura was suspended by the apertures. And these sutures were then stitched on the rim of the dissected temporalis (Figure 7B). When the temporalis was suspended on the plate, it also was sutured with dura, tightly adhering to the plate (Figure 7C).

(5) During suturing incisions, if the tensity of skin allowed, the edge of the skin flap was trimmed to minimize previous scar tissue and to promote wound healing.

POSTOPERATIVE (Blue part in Figure 5):

The postoperative part stressed the management of subcutaneous drainage and wound healing.

**Figure 8:**
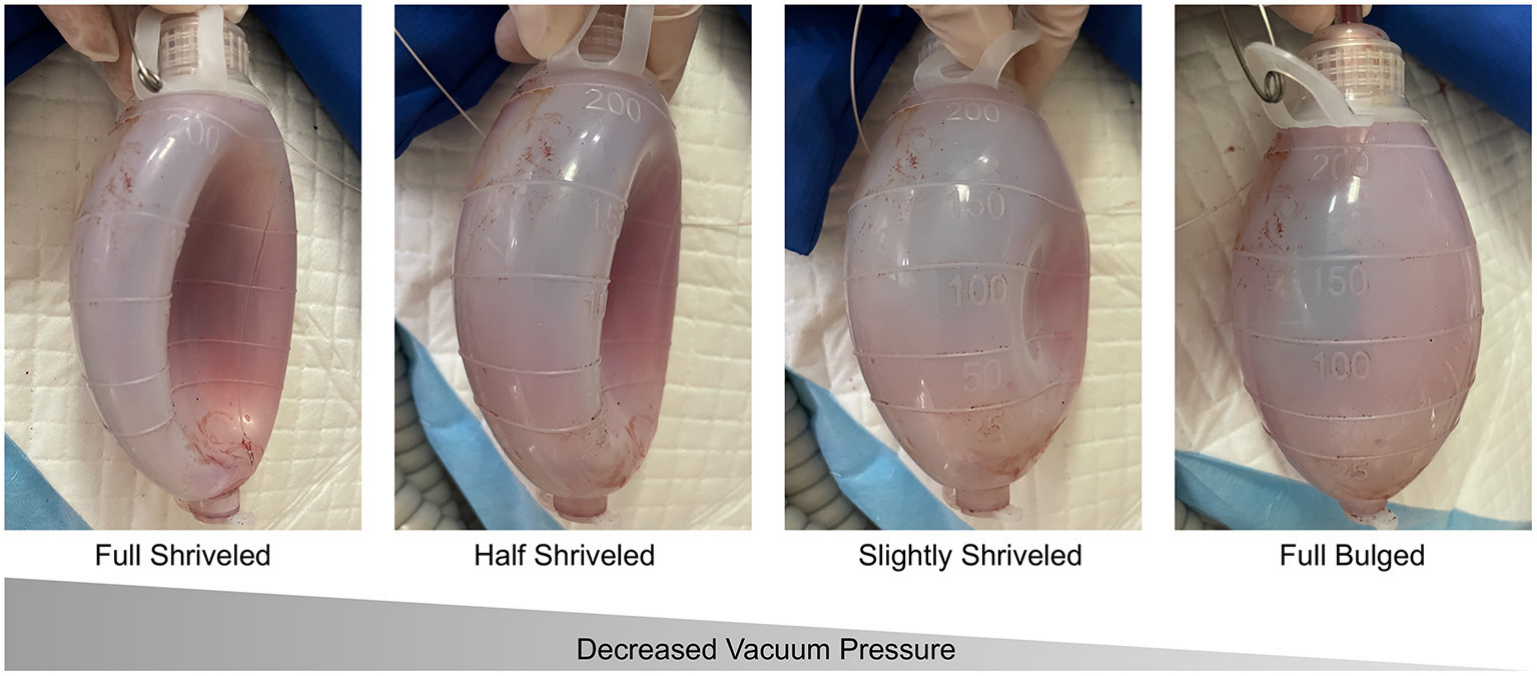
Different levels of shriveled drainage ball.

(1) The duration of subcutaneous drainage was extended to 4 days after surgery.

(2) The vacuum pressure of drainage was evaluated daily and gradually reduced according to the drainage volume and characteristics in the last 24 h. Vacuum balls were used for drainage. The vacuum pressure was controlled by the degree of shriveling of the ball. The state of the ball is classified in order of decreasing vacuum pressure as fully shriveled, half shriveled, slightly shriveled, and fully bulged (Figure 8). Typically, if the volume in the last 24 h was lower than 15 ml, the vacuum pressure was reduced to the next lower level. For patients with cerebrospinal fluid leakage and dura repair during operation, the vacuum pressure was set at the lowest level.

(3) Patients were asked to lie down on the repair side for at least 1 week and transfused additionally to help the brain rebound. Some patients' shunt valves could be adjusted to a high-pressure threshold.

(4) After subcutaneous drainage was removed, the repair side should be tightly warped. When effusion emerged at subcutaneous space (Figures 2A,B), interventions should be immediately taken, e.g., subdural puncture or drainage again. But for effusion only in epidural space and without mass effect (Figure 2C), it could be observed.

(5) Additional nutrition support was implemented to improve wound healing.

Notably, these above managements were not applied in conventional group. In conventional paradigm, preoperative preparations were similar to other elective surgeries. Patients got CT scan to manufacture the plate. Patients were shaved at the day before surgery and got routine laboratory examinations. During surgery, the implanted plate had only 2-mm aperture and perfectly fixed the skull defect. After surgery, the subcutaneous drainage was removed at the 2–4 days postoperatively.

### Statistical Analysis

Statistical analysis was performed using R 4.0.2 (R Foundation, Vienna, Austria). Normally distributed quantitative data are presented as the mean ± standard deviation (SD). Nonnormally distributed quantitative data are presented as the median and interquartile range. The chi-square test or Fisher's exact test were used to analyze differences in incidences between the two groups. The independent *T*-test and Wilcoxon rank-sum test were used to analyze parametric and nonparametric discrete variables, respectively. Statistical inference was conducted at a significance level of 0.05.

## Results

### General Information of Patients

From February 2017 to August 2020, a total of 126 patients underwent PEEK cranioplasty. Patients with a history of previous autologous bone cranioplasty were excluded. A total of 104 patients with complete data were enrolled in the study. Thirty eight patients received conventional perioperative management and were implanted with the conventional plate. After September 2018, a total of 66 patients received the comprehensive treatment paradigm. The demographic and craniectomy-related information of these patients is detailed in Table 1.

**Table 1:** General information of patients received two paradigms.

	**Conventional (*n* = 38)**	**Evolving (*n* = 66)**	***P* value**
Gender			0.40
Male	22 (57.9%)	45 (68.2%)	
Female	16 (42.1%)	21 (31.8%)	
Age (years)	43 ± 17	43 ± 16	0.93
Comorbidities			
Hypertension	9 (23.7%)	9 (13.6%)	0.30
Diabetes	3 (7.9%)	5 (7.6%)	1.00
Seizure	5 (13.2%)	4 (6.1%)	0.38
Hydrocephalus	8 (21.1%)	5 (7.7%)	0.06
Others^#^	6 (15.8%)	8 (12.1%)	0.82
Cause of skull defect			<0.01**
Trauma	21 (55.3%)	51 (77.3%)	
Cerebrovascular diseases	7 (18.4%)	13 (19.7%)	
Tumor	5 (13.2%)	0 (0.0%)	
Diseases of the skull	4 (10.5%)	2 (3.0%)	
Intracranial abscesses	1 (2.6%)	0 (0.0%)	
Site of skull defect			1.00
Lateral	33 (86.8%)	58 (87.9%)	
Bilateral	5 (13.2%)	8 (12.1%)	
Size of skull defect (cm^2^)	56.5 (37.7, 90.53)	75.4 (55.38, 100.14)	0.05
Length of skull defect time (months)	4 (3, 7)	4 (3, 6)	0.26
Sacrificed superficial temporal artery	11 (28.9%)	29 (43.9%)	0.13
Preoperative GCS score	15 (13, 15)	15 (13, 15)	0.65
Preoperative GOSE score	6 (5, 8)	6 (4, 8)	0.74
Folliculitis or irritated scar	13 (34.2%)	25 (37.9%)	0.71

*^#^Including cancer or cancer section history, neurodegenerative disease, heart diseases, tuberculosis history, arrhythmia, thyroid diseases, chronic hepatitis, and gallbladder diseases.*

*GCS, Glasgow Coma Scale; GOSE, Glasgow Outcome Scale Extended.*

*p < 0.05; **p <0.01.

### Incidences of Postoperative Complications in Two Groups With Different Managements

Postoperative complications, including subcutaneous effusion, infection, hematoma, seizure, and implant exposure, were collected and analyzed as the primary outcome in this study (Table 2). In general, the evolving group had a lower total incidence of complications than the conventional group [12 (18.2%) vs. 18 (54.5%), *P* <0.01]. With the evolving treatment paradigm, the percentage of patients with complications was significantly lower than in the conventional group [7 (10.6%) vs. 12 (31.6%), *P* = 0.02]. The evolving group showed fewer infection events; notably, subcutaneous infections, the worst of the possible postoperative infections, were completely prevented in the evolving group [3 (7.9%) vs. 0 (0.0%), *P* = 0.04]. The incidence of postoperative epidural hematoma was also decreased in the evolving group [6 (15.8%) vs. 2 (3.0%), *P* = 0.04]. The percentage of patients with newly emerged seizures was lower in the evolving group than in the conventional group. In total, two implants were removed from patients in the conventional group. For one patient, the implant was removed because of a large exposure; the other patient's implant was removed due to repeated subcutaneous effusion complicated with serious subcutaneous infection.

**Table 2:** Incidence of postoperative complications in two paradigms.

	**Conventional (*n* = 38)**	**Evolving (*n* = 66)**	***P* value**
Postoperative complication	18 (47.4%)	12 (18.2%)	<0.01**
Subcutaneous effusion	12 (31.6%)	7 (10.6%)	0.02*
Epidural hematoma	6 (15.8%)	2 (3.0%)	0.04*
Subcutaneous infection	3 (7.9%)	0 (0.0%)	0.04*
Incision infection	3 (7.9%)	1 (1.5%)	0.14
New seizure	2 (5.3%)	2 (3.0%)	0.62
Implant exposure	1 (2.6%)	0 (0.0%)	0.37
Implant removal	2 (5.3%)	0 (0.0%)	0.13

*p < 0.05; **p < 0.01.

## Discussion

Many studies have focused on comparing various cranioplasty materials with respect to complications (12, 13, 19), shaping effects (20), and total cost (17). No report, however, has sought the interventions to improve postoperative outcomes. In addition, current perioperative managements for cranioplasty are mostly based on each surgeon's practice. In this “Methods” article, we summarized and evaluated a comprehensive perioperative paradigm for PEEK cranioplasty which was mainly developed to prevent postoperative complications. By comparing patients receiving conventional managements, our evolving managements indicated lower incidences of postoperative complications in our clinical practice, including subcutaneous effusion, epidural hematoma, and subcutaneous infection.

Managements of epidural collections and care of wound condition were the highlighted essences in our perioperative paradigm.

Effusion and hematoma were the common epidural collections after PEEK cranioplasty and have drawn considerable research interest (13, 14, 16, 19, 21, 22). Managements of epidural collections in our paradigm aimed to minimize the empty epidural space, which included the specific body position, intraoperative handling dura, and temporalis, modifications on PEEK plate, and the management of subcutaneous drainage.

The specific resting body position was an essential part of this evolving comprehensive paradigm. The preoperative body position recommended for the patient was chosen to adjust the dilation of brain tissue to better fit the plate, which also contributed to decrease empty epidural space. The choice of postoperative body position aimed to maximize brain tissue rebound to shrink the space between the epidural and plate.

Carefully handling dura and suturing temporalis during the operation were the essential surgical procedure for preventing epidural collections. Preserving and repairing the integrity of dura contributed to preventing the epidural leakage of cerebrospinal fluid. The empty epidural space was considered as a probable mechanism for emerging epidural collections (21). And the smooth surface of the PEEK plate makes dura difficult to adhere to. Thus, the complex, but tight suture between dura and plate, between plate and temporalis, anatomically eliminated the empty space beneath and above the plate. After the operation, the pressurized warp at the repair side also aimed to minimize the empty space and to promote the scalp to adhere to the plate.

The modifications of the PEEK plate were innovatively introduced to better conduct the space between subcutaneous and epidural, with the additional larger (4-mm) apertures and the gap around temporalis. According to finite element mechanical analysis, these modifications did not compromise the strength of the PEEK plate, ensuring the safety (see Supplementary Material). We also intentionally extended subcutaneous drainage and gradually decreased the vacuum pressure to promote the fitting of the skin flap and to reduce the epidural empty space. Along with the modified PEEK plate, epidural collections could be more efficiently and completely drained through the enlarged apertures. The larger aperture on the PEEK plate might allow a better flow of vacuum pressure and help the brain tissue rebound, reducing the empty epidural space postoperatively.

In the conventional group, 15.8% of patients presented with epidural hematoma. With the evolving paradigm, this incidence was significantly reduced to 3.0%. And 31.6% of patients presented with subcutaneous effusion in the conventional group, higher than that reported in another study (13, 16, 19). This higher incidence may be attributable to the higher sensitivity of diagnosis from CT images. The evolving paradigm decreased the incidence of subcutaneous effusion to 10.6% with the same diagnosis standard. Notably, among these seven patients in the evolving group who presented with complications, six achieved full recovery, and one experienced recurrence that did not affect daily life.

Postoperative infection is another common and undesirable complication for patients (12, 15, 17, 18). In a multicenter study by Rosenthal et al. 5 patients underwent reoperation to remove the implant because of a serious infection (11). And the implant exposure was the most unfavorable incision-related complication after cranioplasty. Thus, in our perioperative paradigm, we developed preoperative interventions for undesirable scars, sufficient nutritional support, customized design of incision, and intraoperative trimming of incision, to prevent incision-related complications.

The preoperative scalp preparation in our paradigm seems aggressive. But, according to the general patient information, 68.2% of the cranioplasty patients were male, and most were middle-aged. Because the majority of these patients might present with folliculitis, particularly around previous scars (Figure 6). Sterilizing the skin in advance and applying topical antibiotics could effectively prevent the migration of superficially colonized pathogens, which largely decreased the risk of postoperative infections (23).

Scalp blood supply around the incision was emphasized in our perioperative paradigm. A poor scalp blood supply has been linked to undesirable wound healing (24, 25), ultimately resulting in wound infection or implant exposure in the long term. Before surgery, the incision of most patients was designed along the previous craniectomy scar. However, for patients with complicated scars, especially patients with a history of bilateral craniectomy or V-P shunt, the cranioplasty incision may form a site of skin surrounded by scars. Therefore, it is necessary to carefully redesign the incision to protect the scalp blood supply. Moreover, trimming scar tissue and nutritional support were also highlighted to promote wound healing.

In our center, 9.1% of patients in the conventional group presented with postoperative infection, lower than in previous studies (12, 13). With the evolving paradigm, the percentage of patients who experienced infection was reduced significantly. Meanwhile, though statistical support is lacking, our data also revealed a decreased incidence of implant exposure.

Besides the major items involving managing epidural collections and wound healing, our perioperative paradigm also included the managements focusing on brain protection and postoperative aesthetic. The CT perfusion or angiography revealed the cerebrovasculature changes after trauma or hemorrhage. It's of great valuable recommended as a preoperative examination but also depends on the allowance of local policy or health insurance. In this study, we found 4 patients in the conventional group and 7 patients in the evolving group had abnormalities in the vasculature (e.g., aneurysms or angiostenosis). And 2 patients in the conventional group and 3 patients in Evolving group had a high risk of rupture. Thus, they received cerebrovascular intervention before cranioplasty, preventing the risk of postoperative hemorrhage. During operation, the protection of dura and limited usage of electrocautery also reflected the brain protection in this secondary surgery, which may also contribute to the reduced incidence of new-onset seizures in the evolving group (26, 27).

The initial surgery of the brain may bring a psychological burden to patients. Before cranioplasty, patients would have great anxiety or expectation for the surgical outcomes. Therefore, psychological support before surgery was proposed as a necessity in our paradigm. Moreover, suspending temporalis and minimizing scar tissue was also out of considering a better aesthetic outcome. These all parts make our perioperative paradigm comprehensive.

In this study, we primarily developed a comprehensive perioperative paradigm to prevent various risk factors for complications after PEEK cranioplasty. However, this Method article could not provide high-level evidence for clinical practice due to its retrospective design. Meanwhile, the biases from heterogeneity of participants and surgeons' proficiency within the 3 years were also unavoidable in this retrospective study. Future multicenter and prospective study was hoped to provide more information. But it still presented effective interventions improving patient outcomes, which made it valuable to conduct further comparative and prospective research. These managements we presented were based on our data and local health system, e.g., advanced shaving, CTP, and extended drainage. But these managements could be feasible, e.g., the preoperative preparation could be completed before admission. Specific revisions based on regional health/insurance policy are necessary before being adopted by other centers. Importantly, this study provides a comprehensive paradigm as a template for cranioplasty with PEEK, from preoperative preparations to intraoperative treatments and postoperative managements. We expect this study to serve as a reference and impetus for future clinical practice and that the included concepts will provide greater benefit to patients.

## Conclusion

We presented a comprehensive perioperative paradigm for cranioplasty with PEEK to prevent postoperative complications. This comprehensive paradigm provides a template in PEEK repair and is of potential value in future clinical practice.

## Data Availability Statement

The original contributions presented in the study are included in the article/Supplementary Material, further inquiries can be directed to the corresponding author.

## Ethics Statement

The studies involving human participants were reviewed and approved by Ethic Committee of Renji hospital. Written informed consent to participate in this study was provided by the participants' legal guardian/next of kin.

## Author Contributions

ZH, YM, CY, and JF contributed to the conception and design of the study. ZH, YM, and CY collected the data and did the statistical analysis. JH and JF performed the surgery and paradigm described in this article. ZH wrote the first draft of the manuscript and prepared the figures together with JH. QM, GG, JJ, and JF provided critical revision of the manuscript. All authors contributed to manuscript revision, read, and approved the submitted version.

## Funding

This study was sponsored by the National Nature Science Foundation of China (grant no. 82071358) and the Shanghai Academic Research Leader (grant no. 21XD1422400).

## Conflict of Interest

The manufacturer of the PEEK implant, Kontour (Xi'an) Medical Technology Co., Ltd., provided the plates in the finite element mechanical analysis, but was not in any way involved in analysis or reporting of this study. The authors declare that the research was conducted in the absence of any commercial or financial relationships that could be construed as a potential conflict of interest.

## Publisher's Note

All claims expressed in this article are solely those of the authors and do not necessarily represent those of their affiliated organizations, or those of the publisher, the editors and the reviewers. Any product that may be evaluated in this article, or claim that may be made by its manufacturer, is not guaranteed or endorsed by the publisher.
