# Supplementary material for: A Perioperative Paradigm of Cranioplasty With Polyetheretherketone: Comprehensive Management for Preventing Postoperative Complications
Source: Front Surg. 2022 Mar 21;9:856743. doi: 10.3389/fsurg.2022.856743 (PMC8977411; doi:10.3389/fsurg.2022.856743)
Supplement: Supplementary file 1 [file Presentation_1.PDF]

Content:

1.Objective 4
2.Research Plan 4
2.1 Research object 4
2.2 Research analysis 4
2.3 Research scheme 5
3.Research Method 5
3.1 Software of FEA 5
3.2 Method of FEA 5
3.2.1 Geometric Model 5
3.2.2 Material 7
3.2.3 Meshing 7
3.2.4 Boundary 7
3.2.5 Load 8
3.2.6 Result output 8
3.3 Mesh convergence analysis 8
4.Research Result 9
4.1 Intracranial pressure only 9
4.2 External load only 14
4.3 Elasticity coefficient and result statistics 20
5.Conclusion 21
6.Reference 22
Appendix 23
Appendix 1 : Test results under intracranial pressure load 23
Appendix 2 : Test results under external load 26

Finite Element Analysis of Polyetheretherketone Bone Plate

1. Objective

According to the Based on the experimental material parameters and the numerical simulation method in the literature "Design, finite element anlysis (FEA), and fabrication of custom titanium alloy cranial implant using electron beam melting additive manufacturing", the mechanical characteristics of PEEK bone plate are analyzed by finite element method, to provide basis for stress analysis of different specifications of bone plate materials.

2. Research Plan

2.1 Research object

One of the models of PEEK bone plate can be divided into different specifications according to plate thickness T1, plate width L1, angle A and hole diameter D. The specific parameters are shown in Table 1.

Table 1 The specific parameters of PEEK bone plate

Model	Specification (mm)				Material	Graphic
	T1 (mm)	L1 (mm)	A (°)	D (mm)		
01	3.6	90	45°	1.9±0.1、(2.0±0.1)×(4.0±0.1)、4.0±0.1	PEEK	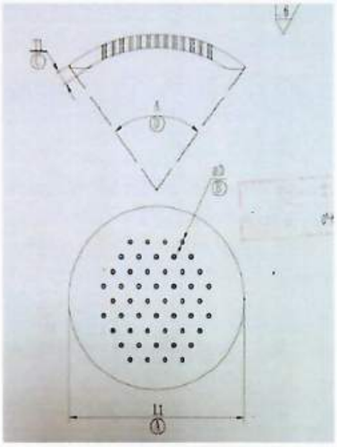

2.2 Research analysis

Polyetheretherketone (PEEK) is a kind of semi-crystalline polymer materials with physical and chemical properties such as high temperature and chemical resistance, and is widely used clinically in cranial bone repair due to its good biocompatibility properties. According to clinical needs and practical applications, PEEK bone plates are often provided with a series of through-holes on the surface to facilitate the lifting of the meninges during surgery and the drainage of post-operative fluid. Due to the fineness of the material, the through-holes in bone plates can result in significant differences in mechanical performance. In order to accurately calculate the mechanical

performance of PEEK bone plates with different apertures in daily life situations, this study will perform finite element analysis on PEEK bone plates with different apertures and angles, so as to provide numerical calculation data reference for clinical medicine.

2.3 Research scheme

According to the above research, the selection scheme is determined as follows:

PEEK bone plates with different apertures were selected for comparative analysis, and 3 models for each aperture within design tolerances included in the calculations, and The case list is shown in Table 2.

Table 2 FEA case list of PEEK bone plate

Case No.	Case (Model- Length -Angle- Hole diameter)		Specification			
			T (mm)	L (mm)	A (°)	D (mm)
1-3	Load 50N/cm^2	Standard round bone plate D2.0±0.1	3.6	90	45	2.0±0.1
4-6		Standard round bone plate D(2.0±0.1)×D(4.0±0.1)	3.6	90	45	(2.0±0.1)×(4.0±0.1)
7-9		Standard round bone plate D4.0±0.1	3.6	90	45	4.0±0.1
10-12	Intracranial pressure 5333Pa	Standard round bone plate D2.0±0.1	3.6	90	45	2.0±0.1
13-15		Standard round bone plate D(2.0±0.1)×D(4.0±0.1)	3.6	90	45	(2.0±0.1)×(4.0±0.1)
16-18		Standard round bone plate D4.0±0.1	3.6	90	45	4.0±0.1

3. Research Method

3.1 Software of FEA

Geometric model processing software: Solidworks2020.

Finite element calculation: ABAQUS 2020.

3.2 Method of FEA

3.2.1 Geometric Model

The PEEK bone plates with different apertures and angles are generated by using Solidworks software through commands such as rotating,excising and stretching. The aperture arrangement was designed according to the actual clinical use. The different calculation models were given the

same material parameters, loads and boundary conditions to analyse the effects of the maximum Mises stress, maximum strain and maximum displacement. Figure 1 shows the model diagrams of standard round bone plate D2.0±0.1, standard round bone plate D(2.0±0.1) x D(4.0±0.1), and standard round bone plate D4.0±0.1.

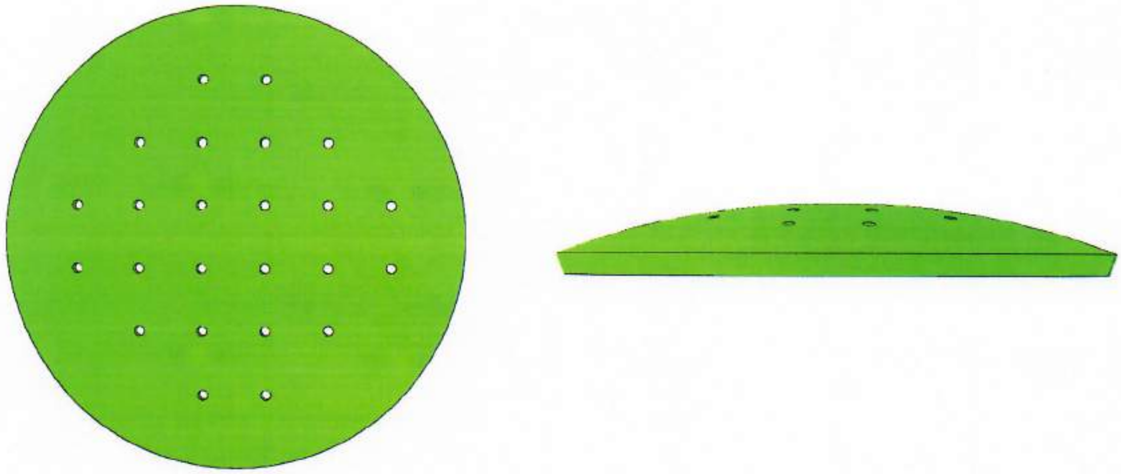

standard round bone plate D2.0±0.1

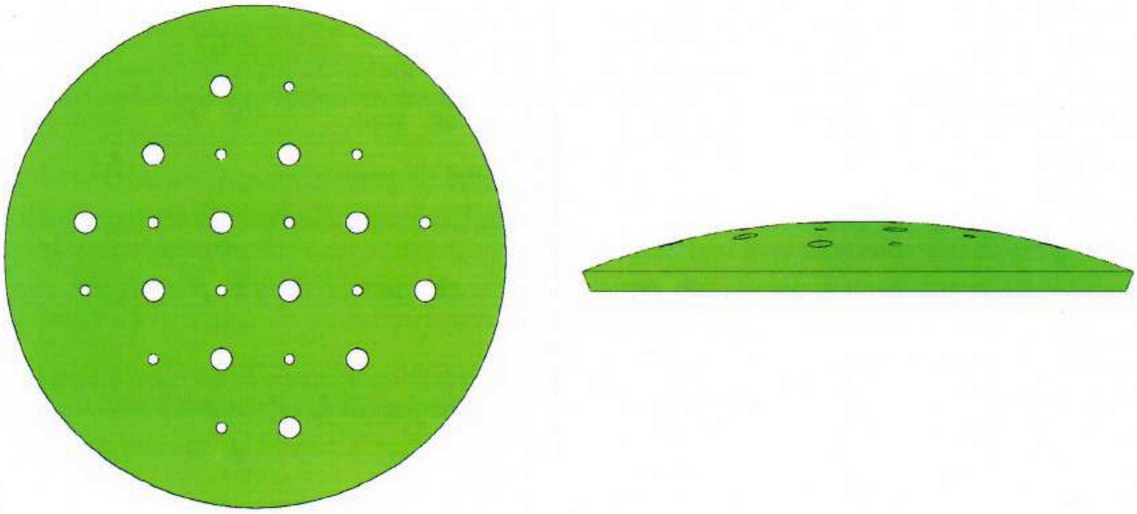

Standard round bone plate D(2.0±0.1) x D(4.0±0.1)

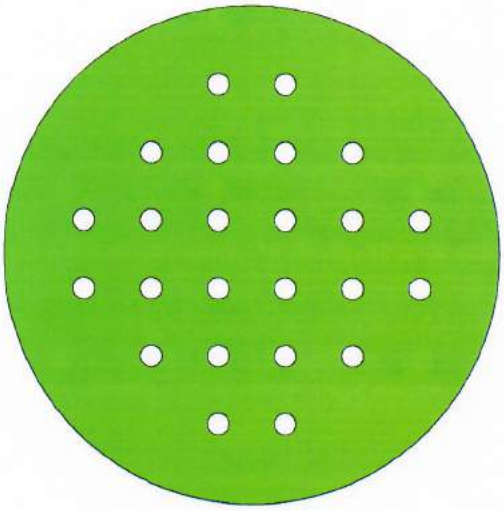

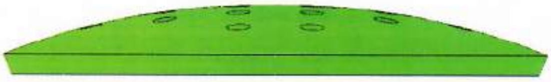

Standard round bone plate D4.0±0.1

Figure1 Model diagrams

3.2.2 Material

According to the material supplier Invbio's inspection bill of materials provided by the customer,The parameters of the PEEK material property were set as follows: Elastic modulus 4400 MPa, Poisson's ratio 0.4, Mass density 1310 kg/cm3, the specific parameters are shown in Table 3.

Table 3 PEEK material property parameters

Property	Value	Unit
Elastic modulus	4400	MPa
Mass density	1310	kg/cm^3
Tensile strength	118	MPa
Compressive strength	192	MPa
Poisson's ratio	0.4	-

3.2.3 Meshing

The principle of meshing is to control the number of meshes based on the accuracy of the calculation, thus reducing the calculation time. In this simulation, the mesh type used for the PEEK bone plate is tetrahedral. After the mesh division is completed, the element quality, number of nodes and elements can be checked.

3.2.4 Boundary

During the use of the PEEK bone plate, the edges of the bone plate are fitted to the original cranial model by means of linking tabs and fixed links with titanium screws, assuming that the links are secure and do not deform, so that fixed restraints are applied at the edges of the bone plate.

3.2.5 Load

Condition 1: "Intracranial pressure >40mmHg is known as severe intracranial pressure increase, with the risk of brain herniation, which is life-threatening", as described in "Advances in the Treatment of Increased Intracranial Pressure and Brain Herniation".The pressure withstand 40mmHg intracranial pressure of PEEK bone plate was analyzed, 40mmHg $\approx$ 5333Pa.

Condition 2: The mechanical performance of the PEEK bone plate in daily life situations is presented in the literature[1], simulating the pressure on the skull when the human body is lying down and resting with the head resting on a pillow. A static force of 50N (0.5MPa) was applied within 1cm^2 of the central area of the PEEK bone plate in the finite element analysis.

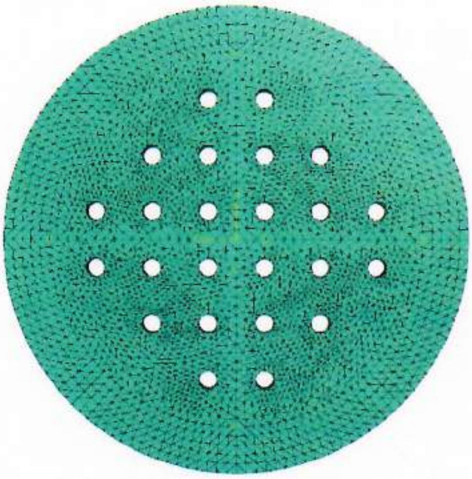

Figure2 Mesh model example

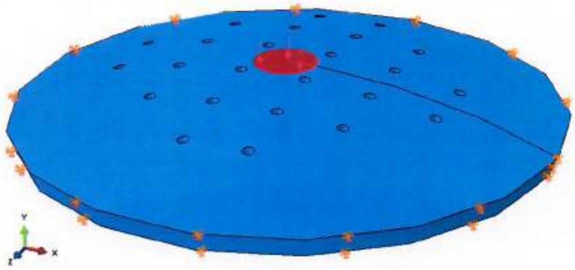

Figure3 Load distribution of FEA model

3.2.6 Result output

Extract the maximum Mises stress, maximum strain and maximum displacement of 16 cases under static load of 50N and intracranial pressure of 40mmHg.

3.3 Mesh convergence analysis

Finite element model mesh convergence analysis: For the case11(Standard round bone plate D1.9), the Peek bone plate model is meshed into 3mm, 2mm and 1.5mm element sizes. The results show that the maximum difference of the maximum Mises stress, strain and displacement are less than 5%. As shown in Table4, the calculation results converge. Therefore, in all calculations, considering the calculation accuracy and speed, the meshing method is to use 2mm element size, and the mesh type is quadratic order tetrahedral element.

$$\text{Deviation} = \frac{|\text{last result} - \text{previous result}|}{\text{last result}} \times 100\%$$

Table4 Mesh Convergence Results

Element Size (mm)	Maximum Mises stress (MPa)	Dev.	Maximum strain	Dev.	Maximum displacement (mm)	Dev.	Elements number
3	0.2923	-	0.00006833	-	0.003033	-	39507
2	0.2904	0.65%	0.00006627	3.1%	0.003042	0.3%	63136
1.5	0.2942	1.30%	0.00006709	1.2%	0.003033	0.3%	96197

4. Research Result

4.1 Intracranial pressure only

According to the conditions of 3.2.5 above, the calculation results of PEEK bone plates with different apertures under impact load are extracted. The maximum Mises stress, maximum strain, maximum displacement and cloud diagram of each model are shown in the table.

Table4.1.1 Calculation results of standard round bone plate D2.0 ± 0.1 under intracranial pressure only

Parameter	Case	Cloud diagrams	Maximum value
Stress (MPa)	Standard round bone D1.9	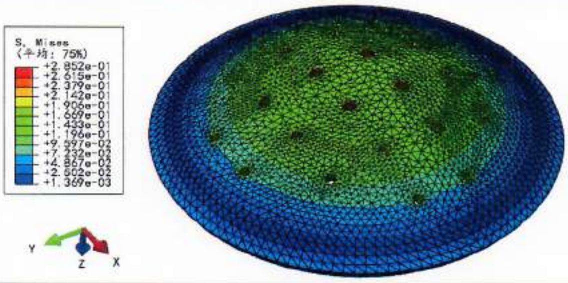	0.2852
	Standard round bone D2.0	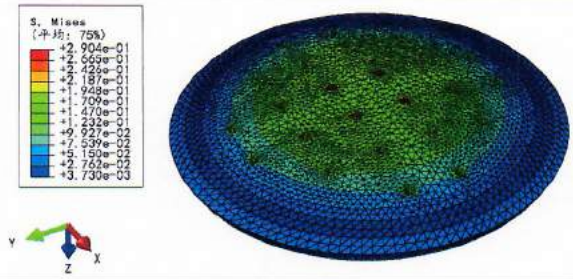	0.2904
	Standard round bone D2.1	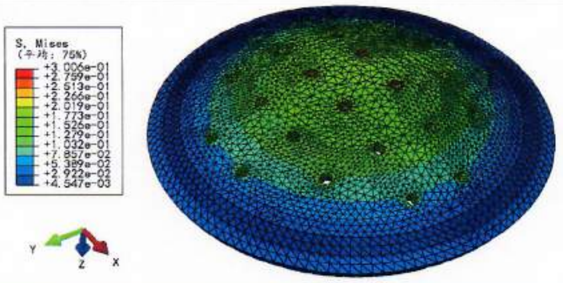	0.3006

[redacted]

Parameter	Case	Cloud diagrams	Maximum value
Strain	Standard round bone D1.9	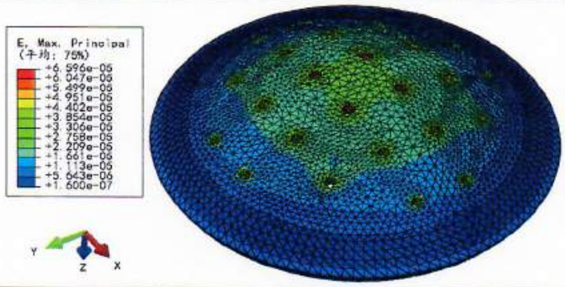	0.00006596
	Standard round bone D2	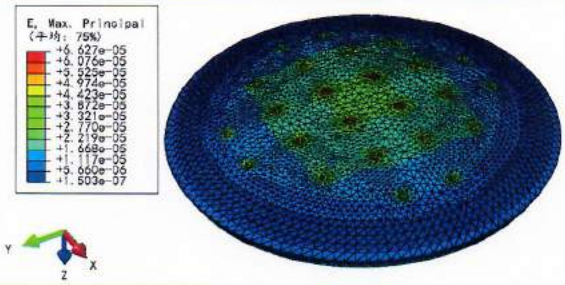	0.00006627
	Standard round bone D2.1	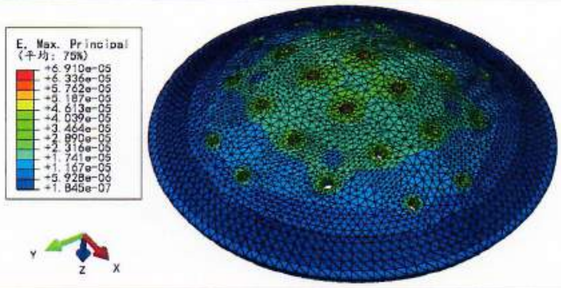	0.00006910
Displacement (mm)	Standard round bone D1.9	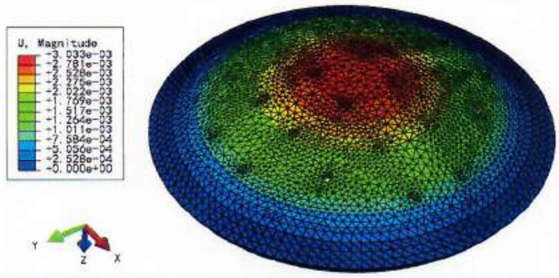	0.003033
	Standard round bone D2	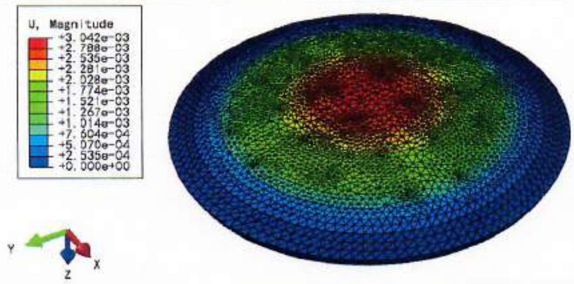	0.003042
	Standard round bone D2.1	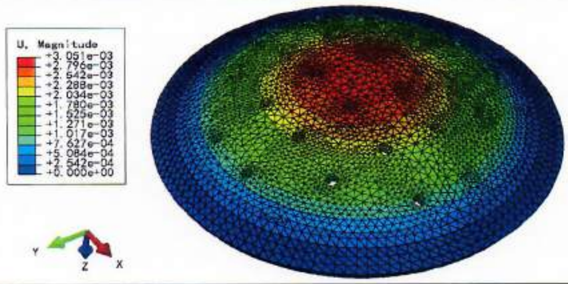	0.003051

Table4.1.2 Calculation results of standard

round bone plate D(2.0±0.1)×D(4.0±0.1) under intracranial pressure only

Parameter	Case	Cloud diagrams	Maximum value
Stress (MPa)	Standard round bone D1.9_3.9	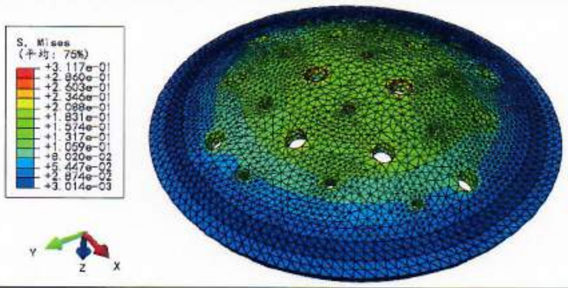	0.3117
	Standard round bone D2_4	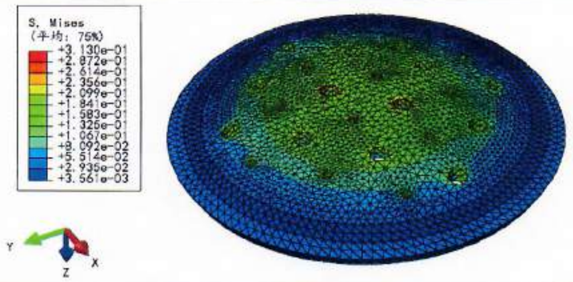	0.3130
	Standard round bone D2.1_4.1	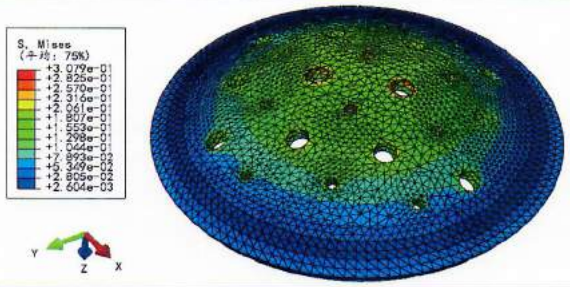	0.3079
Strain	Standard round bone D1.9_3.9	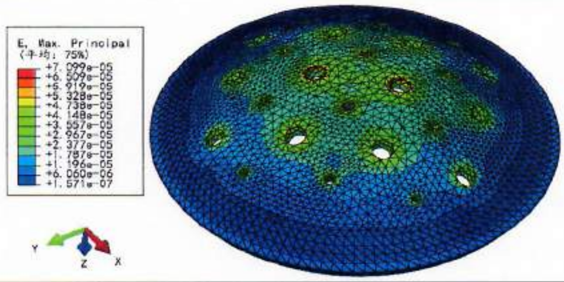	0.00007099
	Standard round bone D2_4	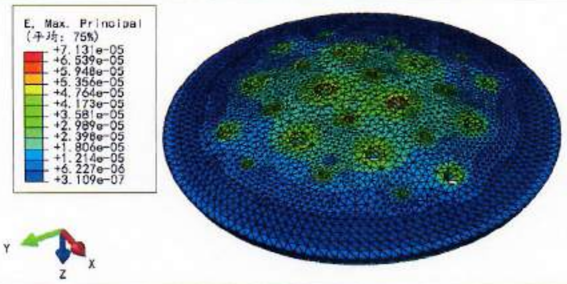	0.00007131

Parameter	Case	Cloud diagrams	Maximum value
	Standard round bone D2.1_4.1	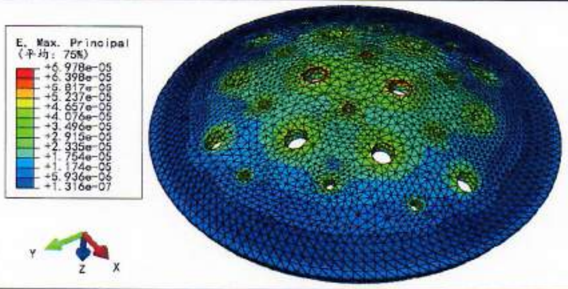	0.00006978
Displacement (mm)	Standard round bone D1.9_3.9	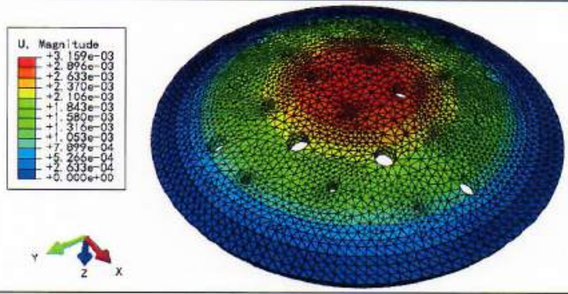	0.003159
	Standard round bone D2_4	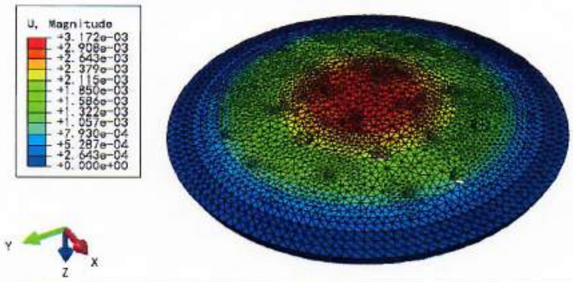	0.003172
	Standard round bone D2.1_4.1	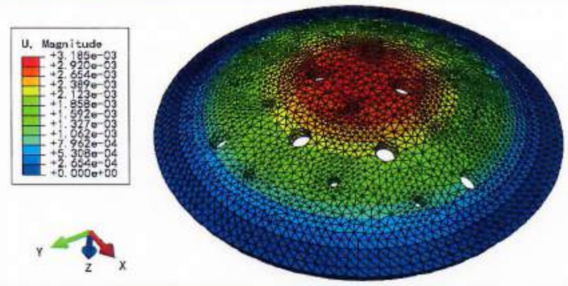	0.003185

Table4.1.3 Calculation results of standard

round bone plate D4.0±0.1 under intracranial pressure only

Parameter	Case	Cloud diagrams	Maximum value
Stress (MPa)	Standard round bone D3.9	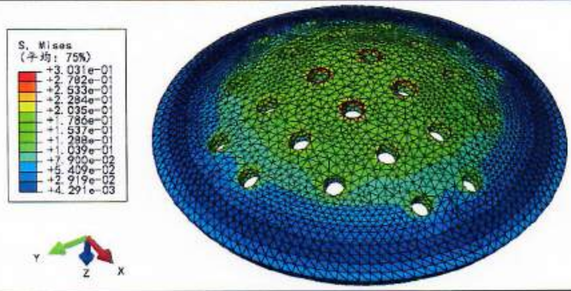	0.3031

Parameter	Case	Cloud diagrams	Maximum value
	Standard round bone D4	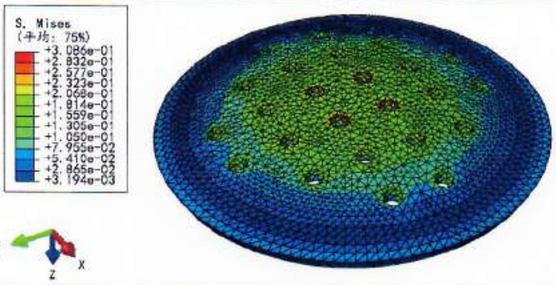	0.3086
	Standard round bone D4.1	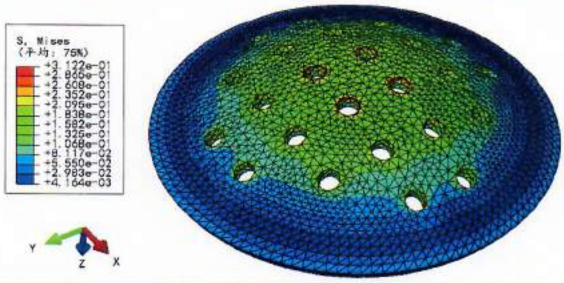	0.3122
Strain	Standard round bone D3.9	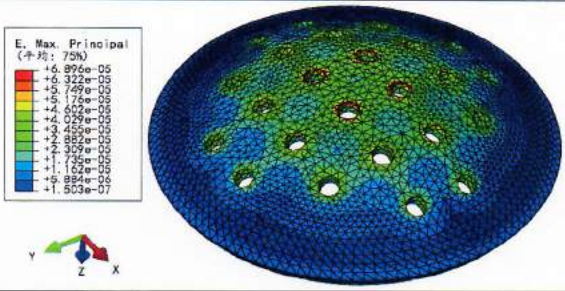	0.00006896
	Standard round bone D4	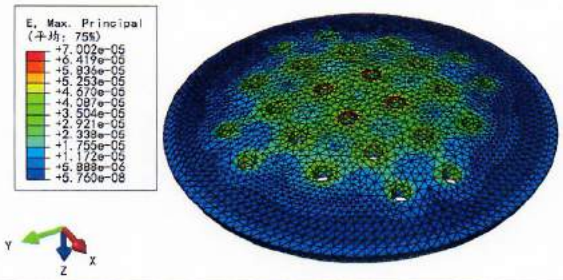	0.00007002
	Standard round bone D4.1	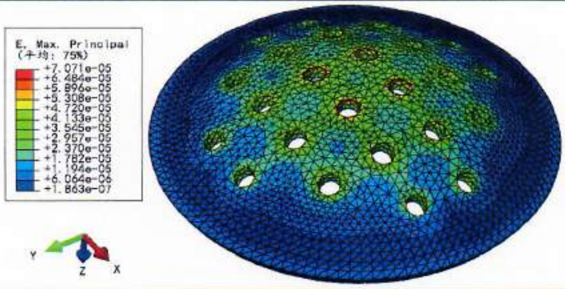	0.00007071

Parameter	Case	Cloud diagrams	Maximum value
Displacement (mm)	Standard round bone D3.9	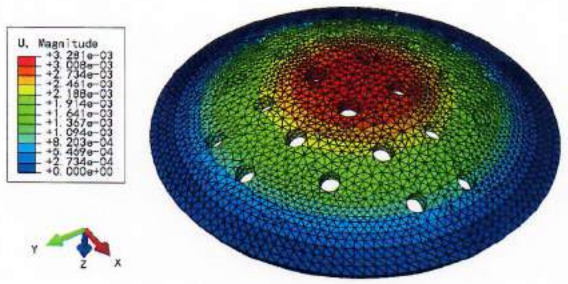	0.003281
	Standard round bone D4	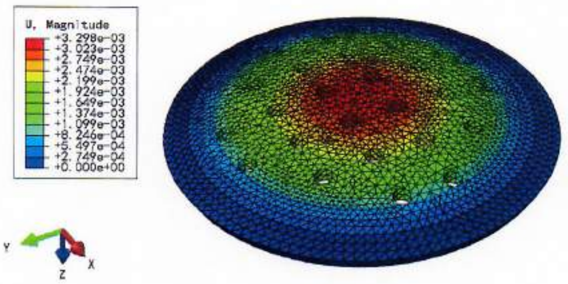	0.003298
	Standard round bone D4.1	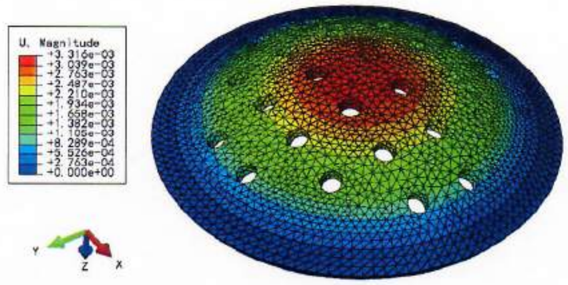	0.003316

Four different models are analyzed by finite element method, and the results output the maximum stress (- VonMises -), the maximum displacement (- Combined displacement -), and the maximum strain (-Equivalent-) respectively. It can be seen from the result cloud chart that the maximum stress and strain occur at the highest convex part of the bone plate of different models, and that the deformation result is the smallest for the same external load when the bone plate aperture is D2.0 ± 0.1, and also the stress is the smallest.

4.2 External load only

According to the conditions of 3.2.5 above, the calculation results of PEEK bone plates with different apertures under external load are extracted. The maximum Mises stress, maximum strain, maximum displacement and cloud diagram of each model are shown in the table.

Table4.2.1 Calculation results of standard round bone plate D2.0±0.1under external load only

Parameter	Case	Cloud diagrams	Maximum value
Stress (MPa)	Standard round bone D1.9	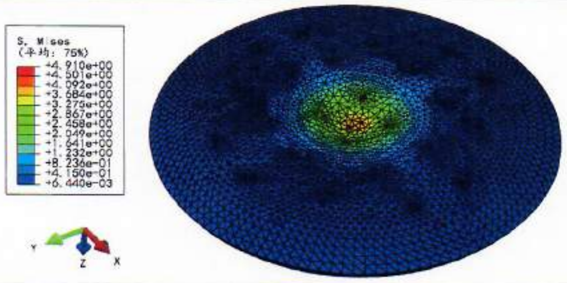	4.910
	Standard round bone D2.0	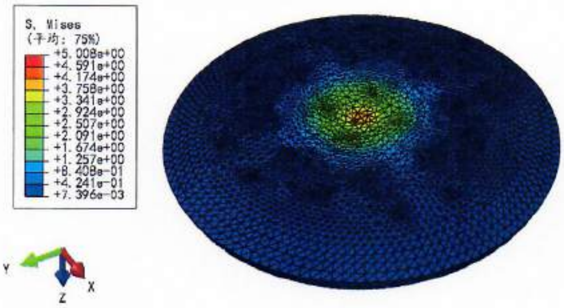	5.008
	Standard round bone D2.1	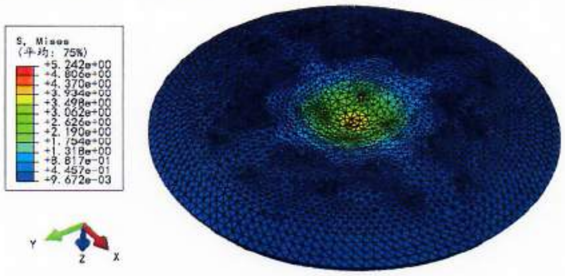	5.242
Strain	Standard round bone D1.9	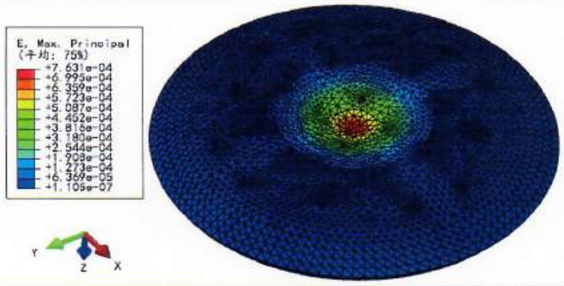	0.0007631
	Standard round bone D2	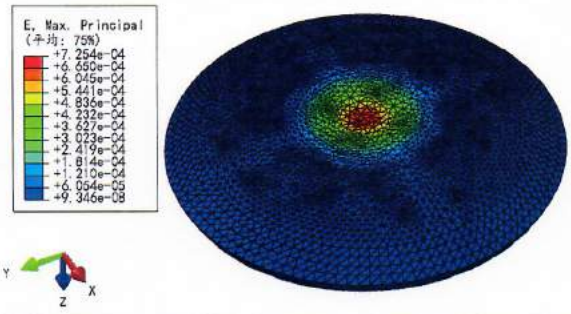	0.0007254

Parameter	Case	Cloud diagrams	Maximum value
	Standard round bone D2.1	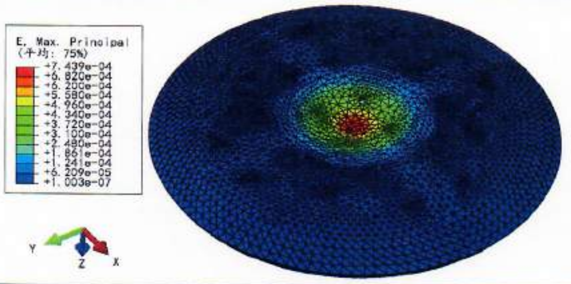	0.0007439
Displacement (mm)	Standard round bone D1.9	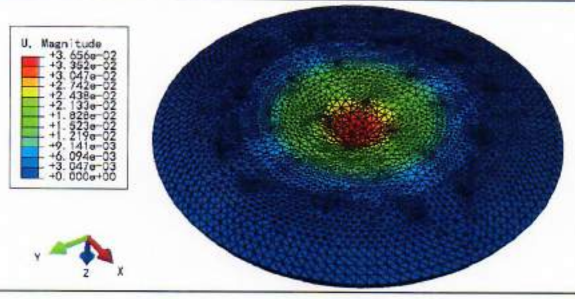	0.03656
	Standard round bone D2	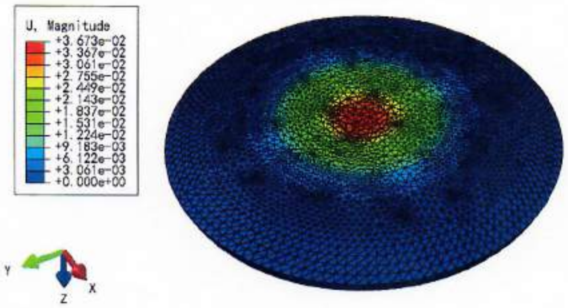	0.03673
	Standard round bone D2.1	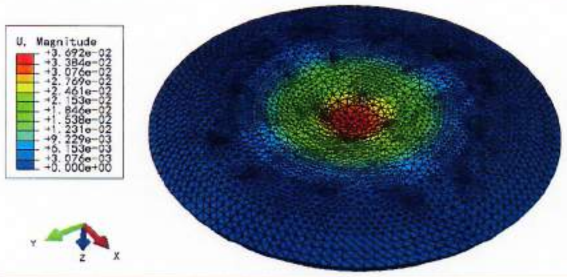	0.03692

Table4.2.2 Calculation results of standard

round bone plate D(2.0±0.1)×D(4.0±0.1)under external load only

Parameter	Case	Cloud diagrams	Maximum value
Stress (MPa)	Standard round bone D1.9_3.9	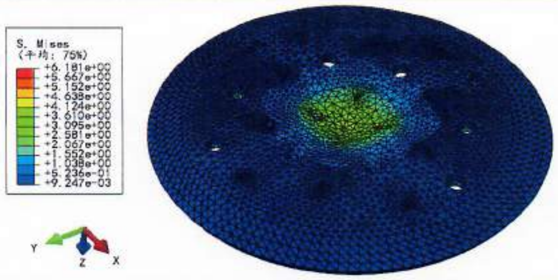	6.181

Parameter	Case	Cloud diagrams	Maximum value
	Standard round bone D2_4	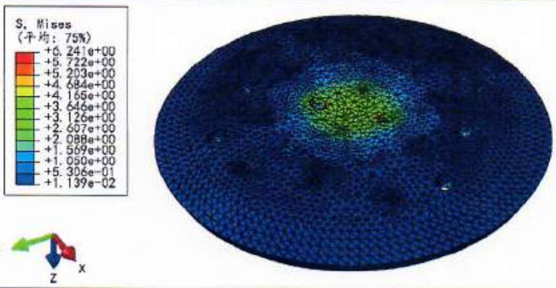	6.241
	Standard round bone D2.1_4.1	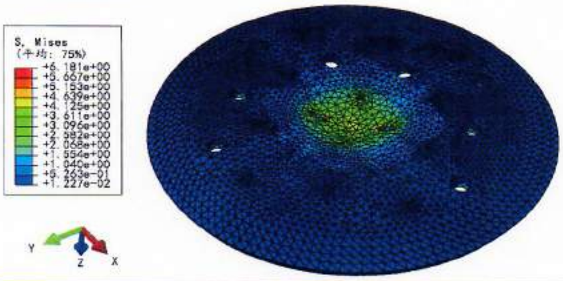	6.181
	Standard round bone D1.9_3.9	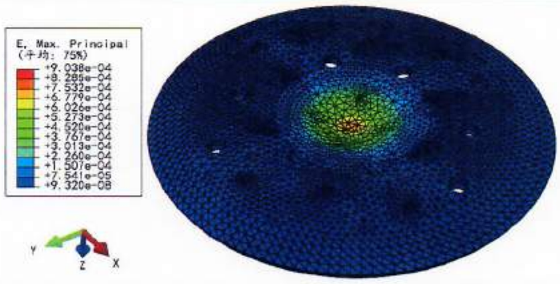	0.0009038
Strain	Standard round bone D2_4	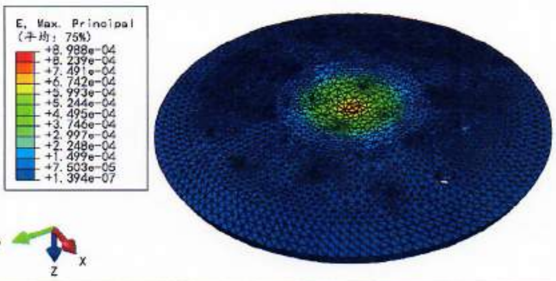	0.0008988
	Standard round bone D2.1_4.1	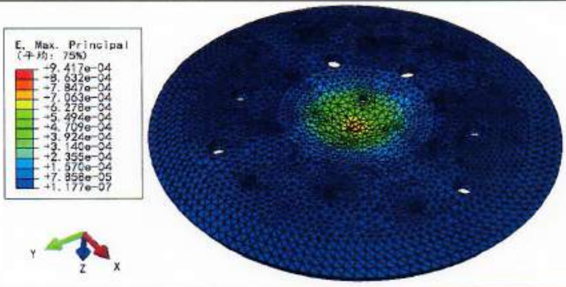	0.0009471
Displacement (mm)	Standard round bone D1.9_3.9	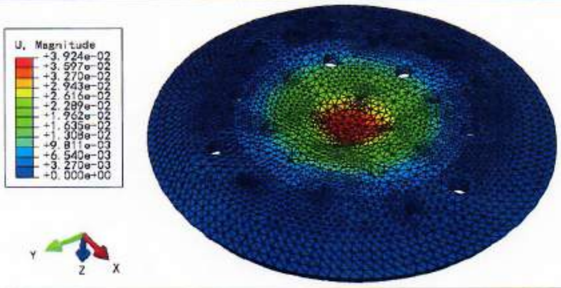	0.03924

Parameter	Case	Cloud diagrams	Maximum value
	Standard round bone D2_4	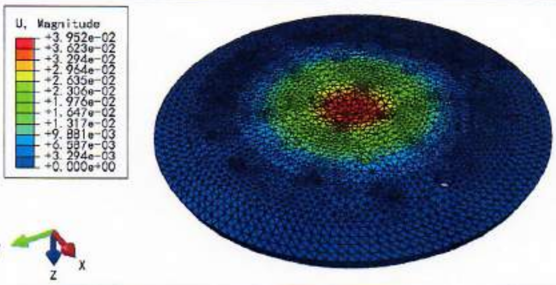	0.03952
	Standard round bone D2.1_4.1	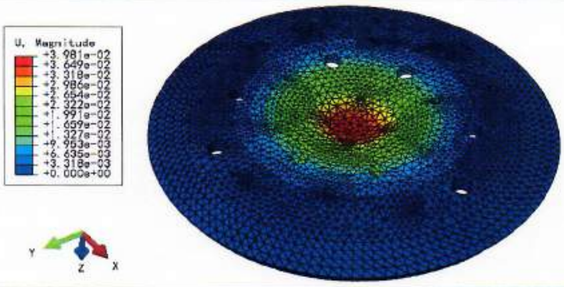	0.03981

Table4.2.3 Calculation results of standard round bone plate D4.0±0.1 under external load only

Parameter	Case	Cloud diagrams	Maximum value
Stress (MPa)	Standard round bone D3.9	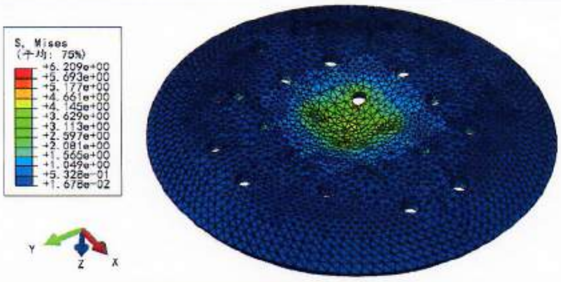	6.209
	Standard round bone D4	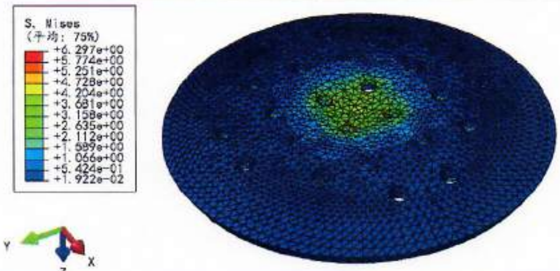	6.297
	Standard round bone D4.1	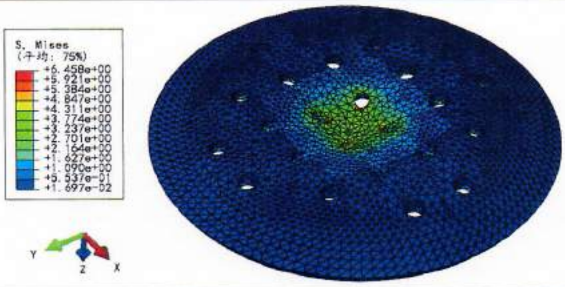	6.458

Parameter	Case	Cloud diagrams	Maximum value
Strain	Standard round bone D3.9	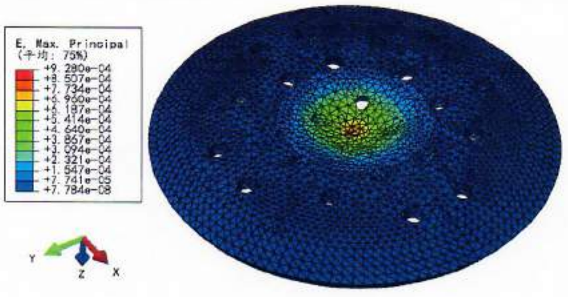	0.0009280
	Standard round bone D4	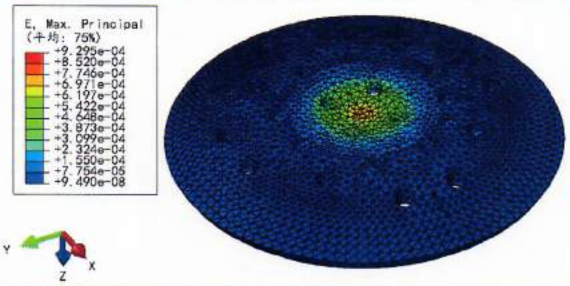	0.0009295
	Standard round bone D4.1	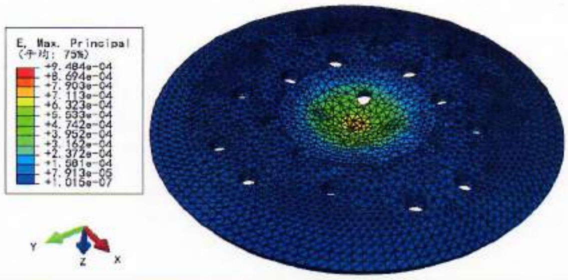	0.0009484
Displacement (mm)	Standard round bone D3.9	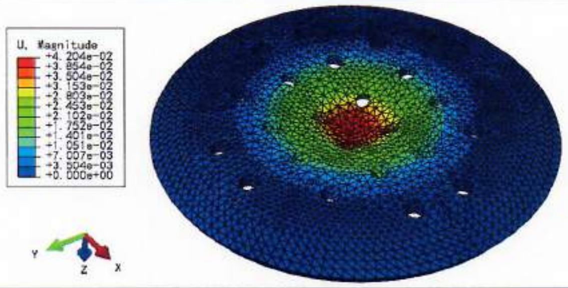	0.04204
	Standard round bone D4	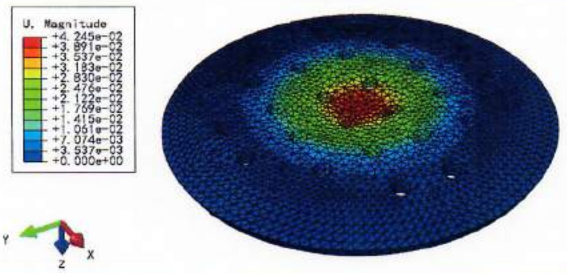	0.04245

Parameter	Case	Cloud diagrams	Maximum value
	Standard round bone D4.1	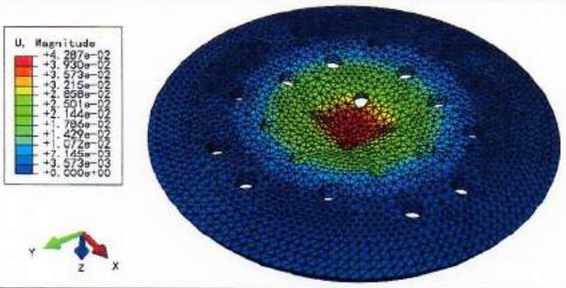	0.04287

Four different models are analyzed by finite element method, and the results output the maximum stress (- VonMises -), the maximum displacement (- Combined displacement -), and the maximum strain (-Equivalent-) respectively. It can be seen from the result cloud chart that the maximum stress and strain occur at the highest convex part of the bone plate of different models, and that the deformation result is the smallest for the same external load when the bone plate aperture is D2.0 ± 0.1, and also the stress is the smallest.

4.3 Elasticity coefficient and result statistics

The ratio of Mises stress to strain is defined as the elasticity coefficient (unit: MPa), and the results are shown in Table 4.3.1 and Table 4.3.2.

Table 4.3.1 Calculation results of each aperture model under intracranial pressure

Aperture	Mises stress (MPa)	Strain	Elasticity coefficient(σ/ε) (MPa)	Displacement (mm)
D1.9	0.2852	0.00006596	4323.83	0.003033
D2.0	0.2904	0.00006627	4382.07	0.003042
D2.1	0.3006	0.0000691	4350.22	0.003051
Mean	0.292	0.00006711	4352.06	0.003042
Standard deviation	0.006	1.41282E-06	4527.48	7.35E-06
D1.9_3.9	0.3117	0.00007099	4390.76	0.003159
D2_4	0.313	0.00007131	4389.29	0.003172
D2.1_4.1	0.3079	0.00006978	4412.44	0.003185
Mean	0.311	7.06933E-05	4397.40	0.003172
Standard deviation	0.002	6.58905E-07	3284.00	1.06E-05
D3.9	0.3031	0.00006896	4395.30	0.003281
D4	0.3086	0.00007002	4407.31	0.003298
D4.1	0.3122	0.00007071	4415.22	0.003316
Mean	0.308	6.98967E-05	4406.03	0.003298
Standard deviation	0.004	7.19738E-07	5199.05	1.43E-05

Table 4.3.2 Calculation results of each aperture model under external load

Aperture	Mises stress (MPa)	Strain	Elasticity coefficient(σ/ε) (MPa)	Displacement (mm)
D1.9	4.91	0.0007631	6434.28	0.03656
D2.0	5.008	0.0007254	6903.78	0.03673
D2.1	5.242	0.0007439	7046.65	0.03692
Mean	5.053	0.000744133	6790.90	0.036736667
Standard deviation	0.139	1.53918E-05	9048.79	0.000147045
D1.9_3.9	6.181	0.0009038	6838.90	0.03924
D2_4	6.241	0.0008988	6943.70	0.03952
D2.1_4.1	6.181	0.0009471	6526.24	0.03981
Mean	6.201	0.000916567	6765.47	0.039523333
Standard deviation	0.028	2.16866E-05	1304.23	0.000232713
D3.9	6.209	0.000928	6690.73	0.04204
D4	6.297	0.0009295	6774.61	0.04245
D4.1	6.458	0.0009484	6809.36	0.04287
Mean	6.321	0.0009353	6758.62	0.042453333
Standard deviation	0.103	9.28332E-06	11105.91	0.000338854

Test results: The statistical software SPSS was used to analyze the significance of the difference in the result data, and the statistical results are shown in Appendix 1 and Appendix 2.

Results analysis: From the test results, it can be seen that when the bone plate aperture is D2.0, under the same boundary conditions and external load, the maximum stress, maximum strain and maximum deformation displacement of the bone plate are significantly smaller than those of the bone plate with 2&4mm aperture and the bone plate with 4mm aperture, at this time, the bone plate has the highest strength and is the least prone be damage. The elasticity coefficient results showed that the difference between the elasticity coefficients of the bone plate with 2mm aperture and the bone plate with 4mm aperture were significant under the intracranial pressure load, while there was no significant difference compared with the bone plate with 2&4mm aperture. There was no significant difference in the elastic coefficients of the bone plate with the three kinds of apertures under external load.

5. Conclusion

When the bone plate aperture is D2.0, under the same boundary conditions and external load, the maximum stress, maximum strain and maximum deformation displacement of the bone plate are significantly smaller than those of the bone plate with 2&4mm aperture and the bone plate with 4mm aperture, at this time, the bone plate has the highest strength and is the least prone be

damage. The elasticity coefficient results showed that the difference between the elasticity coefficients of the bone plate with 2mm aperture and the bone plate with 4mm aperture were significant under the intracranial pressure load, while there was no significant difference compared with the bone plate with 2&4mm aperture. There was no significant difference in the elastic coefficients of the bone plate with the three kinds of apertures under external load.

6. Reference

【1】 Ameen W , Al-Ahmari A , Mohammed M K , et al. Design, finite element analysis (FEA), and fabrication of custom titanium alloy cranial implant using electron beam melting additive manufacturing. 2018.

【2】 Allen, T., Goodwill, S., Haake, S. (2008). Experimental validation of a tennis ball finite-element model for different temperatures, In: Estivalet, M., Brisson, P. (eds.), The Engineering of Sport 7, Springer Nature, Switzerland, 125-133.

[redacted]

Appendix

Appendix 1 : Test results under intracranial pressure load

Intracranial Pressure Load_Descriptives

Variable		N	Mean	Std. Deviation	Std. Error	95% Confidence Interval for Mean		Minimum	Maximum
						Lower Bound	Upper Bound		
Stress	BonePlate_D2	3	.292067	.0078341	.0045230	.272606	.311528	.2852	.3006
	BonePlate_D4	3	.307967	.0045829	.0026460	.296582	.319351	.3031	.3122
	BonePlate_D2&4	3	.310867	.0026502	.0015301	.304283	.317450	.3079	.3130
	Total	9	.303633	.0099590	.0033197	.295978	.311289	.2852	.3130
Strain	BonePlate_D2	3	6.711000E-5	1.730347E-6	9.990162E-7	6.281158E-5	7.140842E-5	6.5960E-005	6.9100E-005
	BonePlate_D4	3	6.989667E-5	8.814949E-7	5.089313E-7	6.770691E-5	7.208642E-5	6.8960E-005	7.0710E-005
	BonePlate_D2&4	3	7.069333E-5	8.069903E-7	4.659161E-7	6.868866E-5	7.269801E-5	6.9780E-005	7.1310E-005
	Total	9	6.923333E-5	1.939240E-6	6.464132E-7	6.774270E-5	7.072396E-5	6.5960E-005	7.1310E-005
Displacement	BonePlate_D2	3	3.042000E-3	9.000000E-6	5.196152E-6	3.019643E-3	3.064357E-3	3.0330E-003	3.0510E-003
	BonePlate_D4	3	3.298333E-3	1.750238E-5	1.010500E-5	3.254855E-3	3.341812E-3	3.2810E-003	3.3160E-003
	BonePlate_D2&4	3	3.172000E-3	1.300000E-5	7.505553E-6	3.139706E-3	3.204294E-3	3.1590E-003	3.1850E-003
	Total	9	3.170778E-3	1.116241E-4	3.720804E-5	3.084976E-3	3.256580E-3	3.0330E-003	3.3160E-003
Coefficient of Elasticity	BonePlate_D2	3	4352.0400	29.16263	16.83705	4279.5960	4424.4840	4323.83	4382.07
	BonePlate_D4	3	4405.9433	10.03008	5.79087	4381.0272	4430.8594	4395.30	4415.22
	BonePlate_D2&4	3	4397.4967	12.96216	7.48371	4365.2969	4429.6965	4389.29	4412.44
	Total	9	4385.1600	30.16907	10.05636	4361.9700	4408.3500	4323.83	4415.22

ANOVA

		Sum of Squares	df	Mean Square	F	Sig.
Stress	Between Groups	.001	2	.000	10.313	.011
	Within Groups	.000	6	.000		
	Total	.001	8			
Strain	Between Groups	.000	2	.000	7.204	.025
	Within Groups	.000	6	.000		
	Total	.000	8			
Displacement	Between Groups	.000	2	.000	265.759	.000
	Within Groups	.000	6	.000		
	Total	.000	8			
Coefficient of Elasticity	Between Groups	5043.224	2	2521.612	6.760	.029
	Within Groups	2238.158	6	373.026		
	Total	7281.382	8			

[redacted]

Post Hoc Tests

Multiple Comparisons

Tukey HSD

Dependent Variable	(I) Hole_Diameter	(J)Hole_Diameter	Mean Difference (I-J)	Std. Error	Sig.	95% Confidence Interval	
						Lower Bound	Upper Bound
Stress	BonePlate_D2	BonePlate_D4	-.0159000*	.0044572	.027	-.029576	-.002224
		BonePlate_D2&4	-.0188000*	.0044572	.013	-.032476	-.005124
	BonePlate_D4	BonePlate_D2	.0159000*	.0044572	.027	.002224	.029576
		BonePlate_D2&4	-.0029000	.0044572	.799	-.016576	.010776
	BonePlate_D2&4	BonePlate_D2	.0188000*	.0044572	.013	.005124	.032476
		BonePlate_D4	.0029000	.0044572	.799	-.010776	.016576
Strain	BonePlate_D2	BonePlate_D4	-2.7866667E-006	9.913365E-7	.069	-5.828359E-6	2.550261E-7
		BonePlate_D2&4	-3.5833333E-006*	9.913365E-7	.026	-6.625026E-6	-5.416406E-7
	BonePlate_D4	BonePlate_D2	2.7866667E-006	9.913365E-7	.069	-2.550261E-7	5.828359E-6
		BonePlate_D2&4	-7.9666667E-007	9.913365E-7	.715	-3.838359E-6	2.245026E-6
	BonePlate_D2&4	BonePlate_D2	3.5833333E-006*	9.913365E-7	.026	5.416406E-7	6.625026E-6
		BonePlate_D4	7.9666667E-007	9.913365E-7	.715	-2.245026E-6	3.838359E-6
Displacement	BonePlate_D2	BonePlate_D4	-2.5633333E-004*	1.111889E-5	.000	-2.904491E-4	-2.222175E-4
		BonePlate_D2&4	-1.3000000E-004*	1.111889E-5	.000	-1.641158E-4	-9.588420E-5
	BonePlate_D4	BonePlate_D2	2.5633333E-004*	1.111889E-5	.000	2.222175E-4	2.904491E-4
		BonePlate_D2&4	1.2633333E-004*	1.111889E-5	.000	9.221754E-5	1.604491E-4
	BonePlate_D2&4	BonePlate_D2	1.3000000E-004*	1.111889E-5	.000	9.588420E-5	1.641158E-4
		BonePlate_D4	-1.2633333E-004*	1.111889E-5	.000	-1.604491E-4	-9.221754E-5
Coefficient of Elasticity	BonePlate_D2	BonePlate_D4	-53.90333*	15.76972	.033	-102.2892	-5.5175
		BonePlate_D2&4	-45.45667	15.76972	.063	-93.8425	2.9292
	BonePlate_D4	BonePlate_D2	53.90333*	15.76972	.033	5.5175	102.2892
		BonePlate_D2&4	8.44667	15.76972	.857	-39.9392	56.8325
	BonePlate_D2&4	BonePlate_D2	45.45667	15.76972	.063	-2.9292	93.8425
		BonePlate_D4	-8.44667	15.76972	.857	-56.8325	39.9392

*. The mean difference is significant at the 0.05 level.

Appendix 2 : Test results under external load

Outside Load_Descriptives

Variable		N	Mean	Std. Deviation	Std. Error	95% Confidence Interval for Mean		Minimum	Maximum
						Lower Bound	Upper Bound		
Stress	Bone Plate_D2	3	5.05333	.170579	.098484	4.62959	5.47708	4.910	5.242
	Bone Plate_D4	3	6.32133	.126271	.072903	6.00766	6.63501	6.209	6.458
	Bone Plate_D2&4	3	6.20100	.034641	.020000	6.11495	6.28705	6.181	6.241
	Total	9	5.85856	.615622	.205207	5.38535	6.33176	4.910	6.458
Strain	Bone Plate_D2	3	7.441333E-4	1.885108E-5	1.088368E-5	6.973046E-4	7.909620E-4	7.2540E-004	7.6310E-004
	Bone Plate_D4	3	9.353000E-4	1.136970E-5	6.564297E-6	9.070561E-4	9.635439E-4	9.2800E-004	9.4840E-004
	Bone Plate_D2&4	3	9.165667E-4	2.656056E-5	1.533475E-5	8.505866E-4	9.825468E-4	8.9880E-004	9.4710E-004
	Total	9	8.653333E-4	9.287699E-5	3.095900E-5	7.939418E-4	9.367249E-4	7.2540E-004	9.4840E-004
Displacement	Bone Plate_D2	3	3.673667E-2	1.800926E-4	1.039765E-4	3.628929E-2	3.718404E-2	3.6560E-002	3.6920E-002
	Bone Plate_D4	3	4.245333E-2	4.150100E-4	2.396062E-4	4.142239E-2	4.348428E-2	4.2040E-002	4.2870E-002
	Bone Plate_D2&4	3	3.952333E-2	2.850146E-4	1.645533E-4	3.881532E-2	4.023135E-2	3.9240E-002	3.9810E-002
	Total	9	3.957111E-2	2.490042E-3	8.300141E-4	3.765710E-2	4.148513E-2	3.6560E-002	4.2870E-002
Coefficient_of_Elasticity	Bone Plate_D2	3	6794.9033	320.37455	184.96833	5999.0488	7590.7578	6434.28	7046.65
	Bone Plate_D4	3	6758.2333	60.98701	35.21087	6606.7332	6909.7335	6690.73	6809.36
	Bone Plate_D2&4	3	6769.6133	217.18355	125.39098	6230.0995	7309.1272	6526.24	6943.70
	Total	9	6774.2500	196.58640	65.52880	6623.1403	6925.3597	6434.28	7046.65

ANOVA

		Sum of Squares	df	Mean Square	F	Sig.
Stress	Between Groups	2.939	2	1.470	95.350	.000
	Within Groups	.092	6	.015		
	Total	3.032	8			
Strain	Between Groups	.000	2	.000	83.979	.000
	Within Groups	.000	6	.000		
	Total	.000	8			
Displacement	Between Groups	.000	2	.000	257.244	.000
	Within Groups	.000	6	.000		
	Total	.000	8			
Coefficient_of_Elasticity	Between Groups	2113.777	2	1056.889	.021	.980
	Within Groups	307055.922	6	51175.987		
	Total	309169.699	8			

[redacted]

Post Hoc Tests

Multiple Comparisons

Tukey HSD

Dependent Variable	(I) Hole_Diameter	(J) Hole_Diameter	Mean Difference (I-J)	Std. Error	Sig.	95% Confidence Interval	
						Lower Bound	Upper Bound
Stress	Bone Plate_D2	Bone Plate_D4	-1.268000*	.101370	.000	-1.57903	-.95697
		Bone Plate_D2&4	-1.147667*	.101370	.000	-1.45870	-.83663
	Bone Plate_D4	Bone Plate_D2	1.268000*	.101370	.000	.95697	1.57903
		Bone Plate_D2&4	.120333	.101370	.502	-.19070	.43137
	Bone Plate_D2&4	Bone Plate_D2	1.147667*	.101370	.000	.83663	1.45870
		Bone Plate_D4	-.120333	.101370	.502	-.43137	.19070
Strain	Bone Plate_D2	Bone Plate_D4	-1.91167E-4*	1.626241E-5	.000	-2.410642E-4	-1.412691E-4
		Bone Plate_D2&4	-1.72433E-4*	1.626241E-5	.000	-2.223309E-4	-1.225358E-4
	Bone Plate_D4	Bone Plate_D2	1.911667E-4*	1.626241E-5	.000	1.412691E-4	2.410642E-4
		Bone Plate_D2&4	1.873333E-5	1.626241E-5	.521	-3.116420E-5	6.863087E-5
	Bone Plate_D2&4	Bone Plate_D2	1.724333E-4*	1.626241E-5	.000	1.225358E-4	2.223309E-4
		Bone Plate_D4	-1.873333E-5	1.626241E-5	.521	-6.863087E-5	3.116420E-5
Displacement	Bone Plate_D2	Bone Plate_D4	-5.71667E-3*	2.520582E-4	.000	-6.490050E-3	-4.943283E-3
		Bone Plate_D2&4	-2.78667E-3*	2.520582E-4	.000	-3.560050E-3	-2.013283E-3
	Bone Plate_D4	Bone Plate_D2	5.716667E-3*	2.520582E-4	.000	4.943283E-3	6.490050E-3
		Bone Plate_D2&4	2.930000E-3*	2.520582E-4	.000	2.156616E-3	3.703384E-3
	Bone Plate_D2&4	Bone Plate_D2	2.786667E-3*	2.520582E-4	.000	2.013283E-3	3.560050E-3
		Bone Plate_D4	-2.93000E-3*	2.520582E-4	.000	-3.703384E-3	-2.156616E-3
Coefficient_of_Elasticity	Bone Plate_D2	Bone Plate_D4	36.67000	184.70876	.979	-530.0672	603.4072
		Bone Plate_D2&4	25.29000	184.70876	.990	-541.4472	592.0272
	Bone Plate_D4	Bone Plate_D2	-36.67000	184.70876	.979	-603.4072	530.0672
		Bone Plate_D2&4	-11.38000	184.70876	.998	-578.1172	555.3572
	Bone Plate_D2&4	Bone Plate_D2	-25.29000	184.70876	.990	-592.0272	541.4472
		Bone Plate_D4	11.38000	184.70876	.998	-555.3572	578.1172

*. The mean difference is significant at the 0.05 level.

—No Content Below—
